# Barriers and Opportunities for WHO ‘Best Buys’ Non-Communicable Disease Policy Adoption and Implementation From a Political Economy Perspective: A Complexity Systematic Review

**DOI:** 10.34172/ijhpm.2023.7989

**Published:** 2024-02-04

**Authors:** Giulia Loffreda, Stella Arakelyan, Ibrahim Bou-Orm, Hampus Holmer, Luke N. Allen, Sophie Witter, Alastair Ager, Karin Diaconu

**Affiliations:** ^1^NIHR Research Unit of Health in Fragility, Institute for Global Health and Development, Queen Margaret University Edinburgh, Musselburgh, UK; ^2^Advanced Care Research Centre, Usher Institute, University of Edinburgh, Musselburgh, UK; ^3^Department of Global Public Health, Karolinska Institutet, Stockholm, Sweden; ^4^Department of Clinical Research, London School of Hygiene and Tropical Medicine, London, UK

**Keywords:** Best Buys, NCD Policy, Complexity Theory

## Abstract

**Background:** Improving the adoption and implementation of policies to curb noncommunicable diseases (NCDs) is a major challenge for better global health. The adoption and implementation of such policies remain deficient in various contexts, with limited insights into the facilitating and inhibiting factors. These policies have traditionally been treated as technical solutions, neglecting the critical influence of political economy dynamics. Moreover, the complex nature of these interventions is often not adequately incorporated into evidence for policymakers. This study aims to systematically review and evaluate the factors affecting NCD policy adoption and implementation.

**Methods:** We conducted a complex systematic review of articles discussing the adoption and implementation of WHO's ‘best buys' NCD policies. We identified political economy factors and constructed a causal loop diagram (CLD) program theory to elucidate the interplay between factors influencing NCD policy adoption and implementation. A total of 157 papers met the inclusion criteria.

**Results:** Our CLD highlights a central feedback loop encompassing three vital variables: 1) the ability to define, (re)shape and pass appropriate policy into law; 2) the ability to implement the policy (linked to the enforceability of the policy and to addressing NCD local burden); 3) ability to monitor progress, evaluate and correct the course. Insufficient context-specific data impedes the formulation and enactment of suitable policies, particularly in areas facing multiple disease burdens. Multisectoral collaboration plays a pivotal role in both policy adoption and implementation. Effective monitoring and accountability systems significantly impact policy implementation. The commercial determinants of health (CDoH) serve as a major barrier to defining, adopting, and implementing tobacco, alcohol, and diet-related policies.

**Conclusion:** To advance global efforts, we recommend focusing on the development of robust accountability, monitoring, and evaluation systems, ensuring transparency in private sector engagement, supporting context-specific data collection, and effectively managing the CDoH. A system thinking approach can enhance the implementation of complex public health interventions.

## Background

 As the burden of non-communicable diseases (NCDs) (such as cardiovascular, diabetes, lung diseases, and cancer) remains high,^1^ governments and their health systems face the increasing challenge of preventing, controlling, and managing chronic disease and care. Estimates suggest that at least 71% of adult deaths in low- and middle-income countries (LMICs) today are due to such diseases.^2,3^ Managing and controlling NCDs and their preventable risk factors — chiefly diet, tobacco, physical inactivity, alcohol, as well as their social determinants — require a coordinated effort to work across sectors.

 In 2011 the World Health Organization (WHO) developed a set of interventions – the so-called best buys recommended for adoption and implementation,^4,5^ being reviewed and expanded in 2023.^6^ The “best buys” options cover the four key risk factors for NCDs (tobacco, harmful use of alcohol, unhealthy diet, and physical inactivity) and the four key disease areas (cardiovascular disease, diabetes, cancer, and chronic respiratory disease). Examples of these “best buys” include increasing excise taxes and prices on tobacco and alcohol purchases; reducing salt through behavior change communication and mass media campaigns, reformulating food products, and front-of-package labelling; physical activity campaigns; and drug therapy and counseling for those who have had a heart attack or stroke.^7^

 A United Nations (UN) General Assembly High-Level Meeting in 2018 and recent research^8^ highlighted that most member states are currently not on track to achieve NCD progress, and evidence points towards stagnation for some of the NCD policies — including “best buys” — implementation. One of the reasons might be that countries face serious challenges in adopting and implementing the series of suggested interventions effectively.^9^ Identifying barriers and facilitators to implementation and adoption and actively addressing these may result in increased uptake and implementation of “best buys,” ultimately addressing Sustainable Development Goal 3.^10,11^ Previous reviews have focused on the cost-effectiveness of “best buys” policy process,^9^ and on specific “best buy” categories and policy types within this (eg, taxation).^12^

 However, there is paucity in terms of political economy and complexity analysis. First, “best buys” are acknowledged to be inherently complex interventions,^13^ that target multiple participants, groups, or organizational levels (population complexity).^14^ Further, the strategies recommended require multifaceted adoption, uptake and integration (implementation complexity), work in a dynamic environment (contextual complexity) and seek to achieve impacts via multiple components (intervention complexity) which are subject to diverse mediators and moderators of effect (pathway complexity). Second, given this underlying complexity and reliance on achieving and sustaining change at multiple levels and within multiple groups, “best buy” implementation must be understood and approached as a political process,^15^ which requires a thorough analysis of the actors, contexts and power dynamics that enable these processes. As such, political economy analysis (PEA)^16,17^ — which focuses on how the allocations of political and economic resources affect who does and gets what, when, and how — can make an important contribution to understanding the current lack of progress behind “best buy” implementation.

 We therefore aimed to identify barriers and facilitators to the adoption and implementation of the NCD “best buys” policies using a complexity and political economy perspective in order to identify cross-cutting themes, as well as similarities and differences by “best buys” category.

 We conducted a systematic literature review, registered in PROSPERO (ID: CRD42020153895). Specifically, our approach incorporated a complexity approach drawing on complexity science and realist review tools, including the elaboration of programme theories and causal loop diagrams (CLDs)^18-20^ and informed by the intervention Complexity Assessment Tool for Systematic Reviews (iCAT_SR) tool.^21^

 The original protocol did not include a complexity perspective which was defined after further consideration of the literature and identifying the most appropriate methods to capture the complex dynamics of NCD policies and interventions.

 Specific research questions were:

 (1) How are “best buys” adoption and implementation conceptualized?

 (2) What are the main barriers and facilitators to the adoption and implementation of the best buys and how do these relate to the conceptualization of adoption and implementation?

 (3) How does context influence “best buys” adoption and implementation, including the presence and absence of specific blocking or enabling factors?

## Methods

###  Information Sources and Searches 

 A systematic search of PubMed, Scopus, Web of Science, WHO IRIS (Institutional Repository for Information Sharing), and Google Scholar databases was conducted in March 2020 and retrieved all studies up to that date, starting from January 2011 (the year when “best buys” were put forward by the WHO). The detailed search strategy is reported in Supplementary file 1.

###  Eligibility Criteria 

 To be included, studies had to be published in peer-reviewed academic journals or be grey literature, report on one or more “best buys” policies targeting population-level change and include an account of adoption and/or implementation and their outcomes from a political economy perspective (ie, analysis of political and economic processes). Policies could be developed at national, regional, and international levels and be implemented in any country or setting. Studies were excluded if they did not explicitly report on adoption or implementation, if they were purely focused on theoretical accounts of adoption and/or implementation without offering empirical evidence on these, and if they were too narrow in scope to address research questions (eg, focusing on cost-effectiveness of “best buys” without discussion of broader political economy elements). A full list of eligibility criteria is available in Supplementary file 2 (Table S1).

###  Study Selection 

 One reviewer (GL) screened titles and abstracts using the above criteria, retaining studies of potential relevance. The same reviewer then screened full texts of all articles, with 20% of full texts independently checked by a second reviewer (SA). Disagreements were resolved by consensus and consultation with a third reviewer when required (KD).

###  Data Extraction 

 An *a priori* study selection and a data extraction template, including information on study characteristics, were developed – based on Cochrane guidelines. Key extraction variables included publication author and year, study design and methods, setting, NCD policy target and policy content or characteristics. We further extracted direct quotations where possible on barriers and facilitators to adoption and implementation, actors involved in the latter processes, and characteristics of the broader context surrounding processes. The data extraction template was piloted on an initial set of 10 included studies, and, once refined, was used by two reviewers (GL and SA) to extract data from retrieved studies.

###  Quality Assessment 

 Given the diversity of study methods employed, we decided to use the modified Mixed Methods Appraisal Tool (MMAT) checklist, which includes specific elements for qualitative, quantitative, and mixed methods studies. Two authors (GL and SA) conducted the quality assessment in relation to (1) clarity of research question/s; (2) appropriateness of data collected to address research questions; (3) appropriateness of study methods; (4) findings adequately derived; (5) interpretations sufficiently backed up by data; and (6) coherence between qualitative data sources, collection, analysis, and interpretation. Additionally, for a subset of recommendations that were considered particularly relevant for the decision-making process and for NCD implementers, we also applied the CERQual (Confidence in the Evidence from Reviews of Qualitative Research).^22^

###  Bibliometric Analyses 

 We conducted bibliometric analyses to describe the included studies, including trends in publications on the topic over time and geographic distribution of studies.

###  Specific Analytic Approaches for Each Research Question

 (1) How are “best buys” adoption and implementation conceptualized?

 Following scoping of the research field and reading of included studies, we elaborated an initial programme theory (depicted as a CLD) (See Box 1) surrounding “best buys” adoption and implementation. This highlights how literature conceptualizes adoption and implementation, including and political economy and contextual factors and anticipated mechanisms of change.

**Box 1. Initial Programme Theory Behind “Best Buys” Adoption and Implementation**

**Figure:**
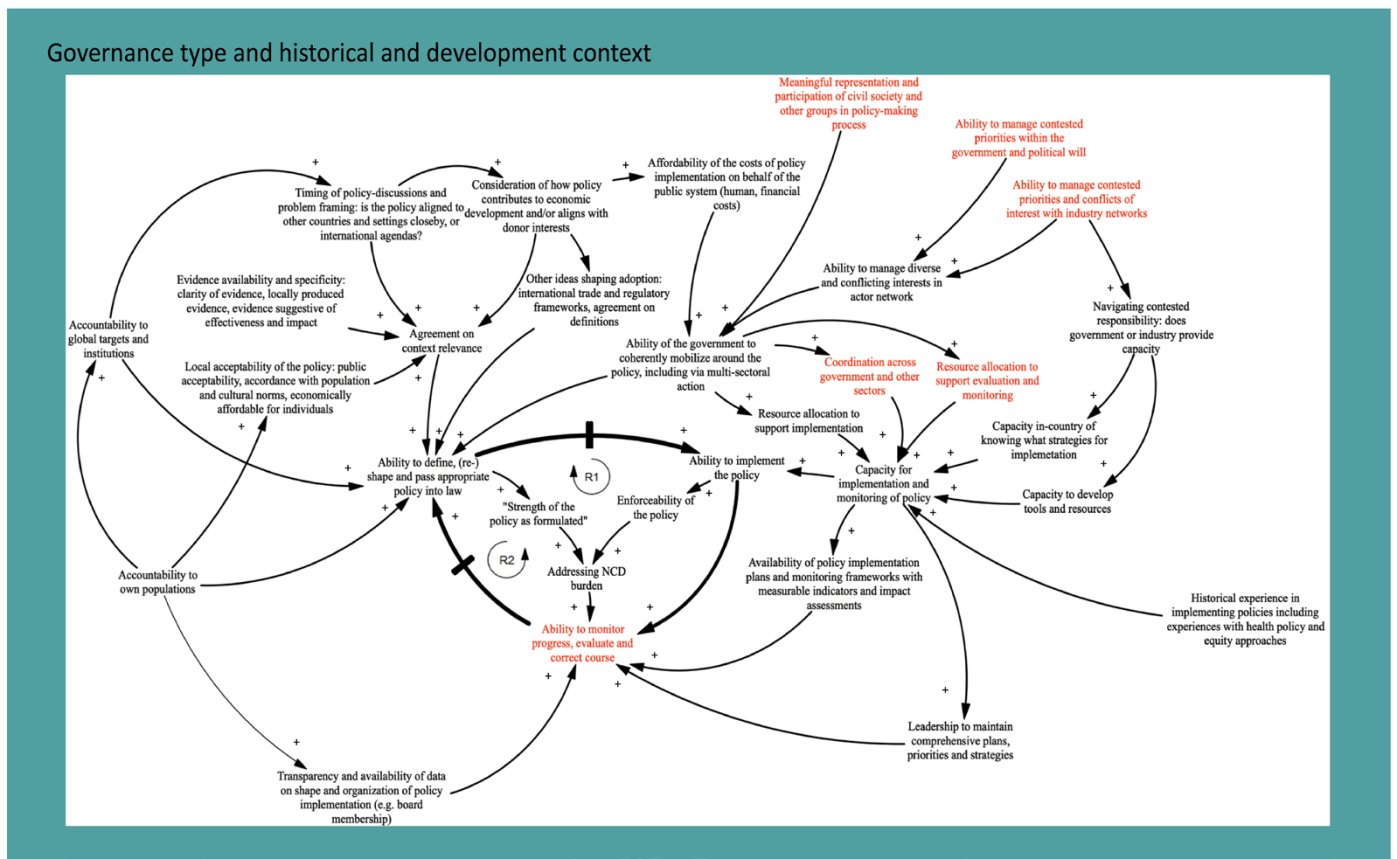

The programme theory highlights a central dynamic (or reinforcing feedback loop, R1) around three main abilities to define and pass, implement and monitor the policy (in red, the specific variables we a-priori believed to constitute facilitators to implementation and confirmed by the review). A second reinforcing feedback loop is indicated as R2.

The above diagram depicts the central processes involved in “best buy” adoption and implementation and the abilities required by the various stakeholders involved in such processes to bring about successful impacts on NCDs.

The diagram conceptualizes adoption as stakeholders working to define, shape and pass an NCD policy into law and further implementing and monitoring this within a policy cycle. The central dynamic (triangle in bold) illustrates the three core abilities we believe various stakeholders should have in order engage in this process.

Specifically, we focus on:

*The ability of stakeholders to define, shape and pass an NCD policy into law (see upper left-hand corner):* We posit that the ability of stakeholders to define and agree on a specific policy is foremost influenced by agreement on context relevance, which in turn depends on the following ideas and interests: the local acceptability of the policy (ie, is the policy accepted by the public), evidence availability and specificity in relation to the policy (ie, is there sufficient context specific evidence or other evidence that supports the policy?), the timing of when policy related discussions occur (ie, is there a window of opportunity either regionally or nationally to pass the policy?) and the consideration of how the policy may impact on economic interests (ie, does passing the policy compromise economic interests of specific parties or is it likely to yield benefits which outweigh potential risks). Further interests shaping how a policy is defined concern governance stakeholders’ accountability to their beneficiary populations and also towards other global institutions (eg, WTO, WHO among others) and wider frameworks and interests which are at play (eg, trade regulations and frameworks or global health targets).

According to how the above ideas and interests pan out and how strong the government’s ability is to mobilize multi- and intersectoral action around the policy, the policy itself may be shaped to be “weak” or “strong” – ie, indicating voluntary measures and targets for other stakeholders to follow, or setting out standards which can be enforced, including by punitive mechanisms. This ability of government is critically dependent on how affordable the policy is to implement, the ability of the government, and particularly the various ministries that may be engaged in policy formulation to agree on a coherent set of priorities, and also the management of potential conflicts of interest between private and public actors (eg, industry and government).

*The ability of the same or other stakeholders to implement said policy (See upper right-hand corner)*: Once a policy is defined and shaped, its impact further depends upon the capacity of various stakeholders to engage in implementation. Specifically, we identify the need to earmark resources (financial and human, including technical expertise) to support implementation and monitoring thereof; this would mean sufficient capacity to support engagement and coordination across multiple sectors. The government’s central ability to mobilize around the policy and make resources and coordination happen cannot be overstated.

*The ability to monitor progress and evaluate policy impacts and as necessary correct course* (See low centre): Policy formulation and implementation should not be viewed as one-time activities and should be understood as part of a policy cycle. Over time, implementation and monitoring should inform how policies may be re-shaped or reformulated in order to improve impacts. Critical to this latter ability is sufficient leadership across whatever policy is defined and then implemented to also ensure adequate follow-up and monitoring and also the availability of concrete and pragmatic monitoring plans, frameworks and relevant data, including benchmarks for specific time-periods against which implementers could be held accountable.

**Implementation Facilitators**

Red points in the CLD mark the specific variables we a-priori believed to constitute facilitators to implementation.

Hypotheses that arise in relation to context specificity and fragility include:

Fragility is likely to mean that contexts have little to no meaningful representation and participation of civil society, accountability to populations and external actors, as well as ability to manage contested priorities around health issues or NCDs in particular – this also may leave open the field for increased industry interference in policy processes, thus compromising the passing of any policy into law and the shape and enforceability of any policy.

Fragility is possible to also lead to diminished financing capacity for implementation and monitoring, as well as compromise availability of human resources who could advise appropriately on the shape of policies and lead on implementation and monitoring.

It is very plausible that there is no local data available for extremely fragile contexts to inform local priority-setting of ‘best buys’ among other policies.

------------------

Abbreviations: NCD, non-communicable disease; WTO, World Trade Organization; WHO, World Health Organization; CLD, causal loop diagram.

(2) What are the main barriers and facilitators to the adoption and implementation of the “best buys” and how do these relate to the conceptualization of adoption and implementation?

 We thematically and narratively synthesized findings across the literature and contrasted these to the initial programme theory, revising and refining this as relevant and highlighting the main barriers and facilitators to “best buys” adoption and implementation.

 (3) How does context influence “best buys” adoption and implementation, including the presence and absence of specific blocking or enabling factors?

 As per our programme theory, and in line with recommendations for reviews adopting a complexity perspective, and the need to account for heterogeneity, we conducted initial analyses and synthesis by taking into account the contextual features of countries/settings as reported in included studies and offer an overview of how these may influence presence and absence of specific barriers or facilitators to “best buys” adoption and implementation. We paid attention to contexts identified as fragile as per the Organisation for Economic Co-operation and Development’s (OECD’s) States of Fragility 2020 report,^23^ given both the socio-political challenges experienced by such contexts and the vulnerability of populations in these settings. OECD defines fragility as the “*combination of exposure to risk and insufficient coping capacities of the state, system and/or communities to manage, absorb or mitigate those risks.”*^24^ The OECD fragility framework is built on five dimensions of fragility — economic, environmental, political, societal, and security — and measures each of these dimensions through the accumulation and combination of risks and capacity.

 Following a PEA, we analysed barriers and facilitators to adoption and implementation in relation to context, actors involved in processes (who engages), mechanisms of engagement (how does engagement occur), and resources and evidence base used for engagement (with what resources and based on what information). The programme theory described below (See Box 1) served as our conceptual framework.

###  Programme Theory Development and Refinement 

 Following scoping of the research topic and based on preliminary assessment of the included studies, reviewers (GL, SA, and KD) elaborated an initial programme theory based on an initial reading of a sub-set of documents summarising current conceptualizations as presented in global literature on “best buys” adoption and implementation. The programme theory takes the shape of a CLD and serves as a mechanism to systematically map out the diverse sources of complexity acknowledged in relation to “best buys” (population, context, intervention and pathway complexity). A ‘seed model’ was initially developed as the central loop to construct and elaborate the programme theory as it is presented in Box 1 We revisited the theory after the review was conducted but minimal changes were made.

 During this process, reviewers adopted a user perspective (ie, considering what issues are of relevance to “best buys” Implementers) and were guided by the “Three Is” PEA framework, which considers the dynamics behind how interests, ideas and institutions shape political processes. This paper is also rooted in the theory of agency and power, two further constructs of relevance when considering “best buys” implementation across settings. The theory thus summarises the varied influences on “best buys” adoption and implementation and anticipated mechanisms of change needed to bring about improved health.

Box 1 explains the programme theory and presents the CLD. The box also (*a*) highlights the factors identified by the review team as likely to be main barriers to “best buys” adoption and implementation, and (*b*) identifies initial hypotheses relating to how these factors may vary according to contextual characteristics.

###  Narrative Synthesis 

 We conducted a narrative synthesis of data extracted and report this in line with synthesis without meta-analysis guidelines.^25,26^

####  Study Grouping

 First, we grouped interventions according to policy type, based on WHO classification of public health/risk factors and health system focus. We thus group retrieved studies according to their focus on: (*i*) diet, (*ii*) alcohol, (*iii*) tobacco, (*iv*) physical activity, and (*v*) health system-related interventions (eg, interventions focused on diabetes, cardiovascular diseases, and cancers). The category of diet included interventions on salt, sugar, obesity, and nutrition policies.

 Second, for each of the above groupings, acknowledging the important role context may play on adoption and implementation, we grouped studies according to the country or setting they refer to. We distinguish:

- Studies discussing non-setting specific policies (eg, studies focused on global overviews);
- Studies on fragile contexts as defined by OECD or by the studies’ authors (eg, Pacific Islands);
- Studies focused on specific countries or settings not deemed fragile by (*ii*).

We decided to adopt this framing as fragility is an increasingly concerning issue in global health and a critical concept to the design and implementation of interventions^27^; further, the initial programme theory (Box 1) highlighted that such settings may face very specific “best buys” adoption and implementation challenges and as such are deserving of a separate study.

####  Synthesis by Policy Type and Context

 Third, for each of the studies grouped as per the above, we then reviewed data extracted and used qualitative thematic comparative analysis to identify themes relating to barriers and facilitators for each of the “best buys” considered for adoption and implementation in each of the above specific contexts. One reviewer (GL) prepared summary tables that offer an overview of these detailed findings; tables were further critically discussed by the research team and predominant themes were derived following group discussion.

####  Synthesis Across Policy Types and Contexts

 Fourth, we proceeded to consider differences and patterns relating to the findings of the above analyses across diverse contexts and also “best buys” policies. At this stage, we offer a narrative descriptive summary of patterns across the literature and synthesise information across all studies reviewed to identify the main barriers and facilitators to “best buys” adoption and implementation overall.

 Finally, we further contrast the themes identified as part of steps 3 and 4 against the initially developed programme theory and CLD and comment on whether the a priori identified barriers and context-related hypotheses hold following analysis of included studies.

## Results

###  Study Characteristics 

**Figure 1:**
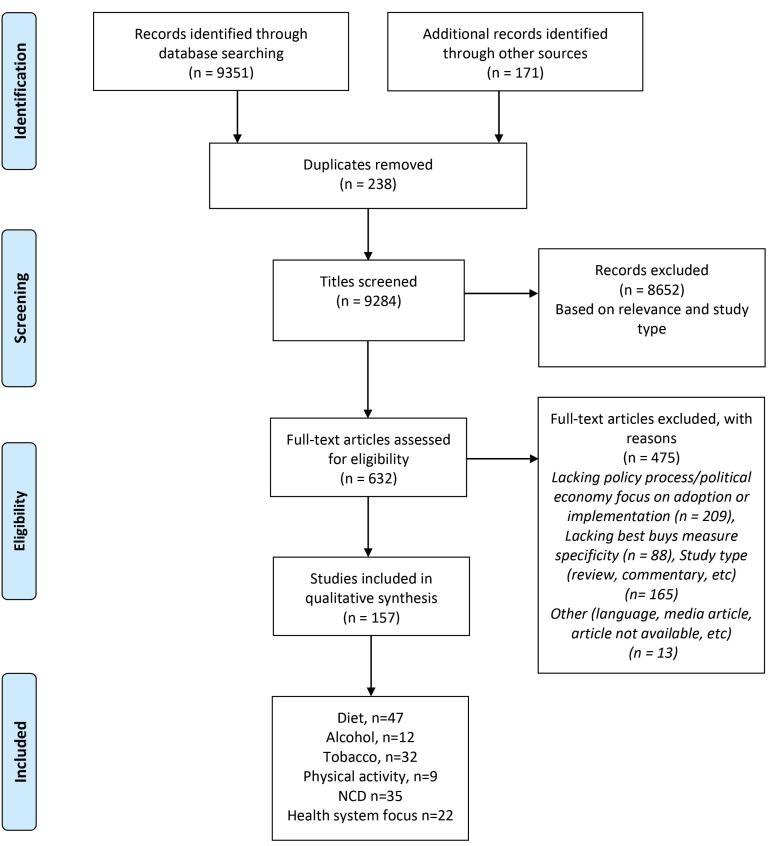
PRISMA Diagram. Abbreviations: PRISMA, Preferred Reporting Items for Systematic Reviews and Meta-Analyses; NCD, non-communicable disease.

**Figure 2:**
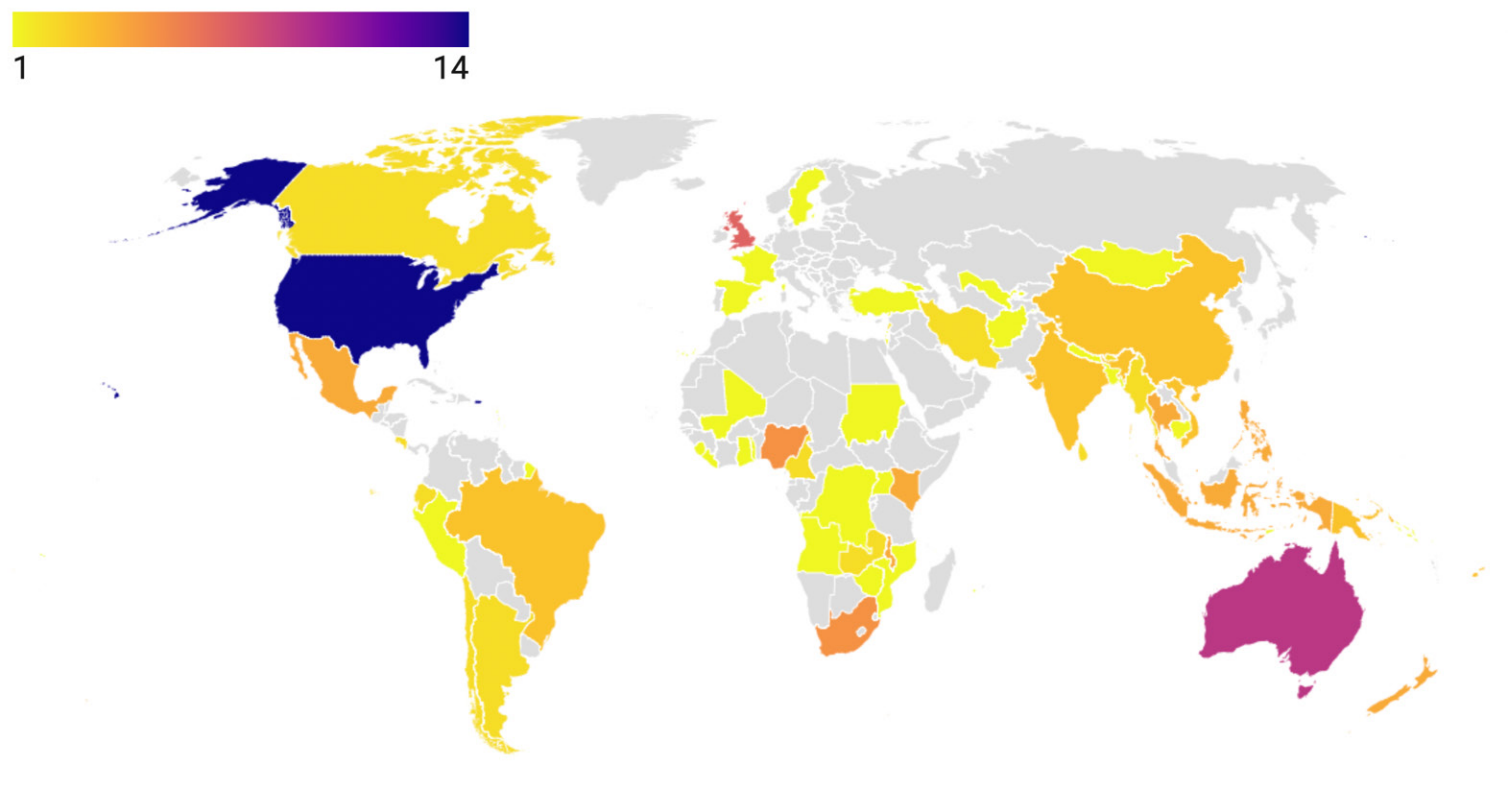
Geographical Distribution of Countries Mentioned in the Studies Under Review.

 Our searches yielded 9237 records. After removing duplicates and studies that did not meet the inclusion criteria, 157 papers were included (Figure 1). Overall, 23 (14%) studies reported information on fragile contexts, 98 (62%) on non-fragile contexts, 9 (0.05%) on both fragile and non-fragile settings, and 27 (17%) had a global focus (or no setting specificity) (Figure 2). The majority of the included studies (124, 79%) used a qualitative approach, including key informant interviews, policy or document analysis, and case studies, among others. The remaining studies used quantitative, mixed method or other study methods (n = 33, 21%). Diet was the most reported area of study (n = 47, 30%) among all NCD policies under review, whereas few studies focused on physical activity and health system related “best buys” (n = 9, 0.05%, n = 22, 14%, respectively). For the list of included studies, see Supplementary file 3 (Table S2).

###  Evidence Synthesis

 A presentation of findings is available in the appendices, with the below sections and Tables 1 and 2 offering an overview. Findings are presented based on the key variables identified per our programme theory (Box 1).

**Table 1:** Summary of Key Review Findings

	**Summary of Key Review Findings**
Tobacco	Legal capacity was a key resource, often not available, to ensure effective adoption and implementation; in this regard FCTC was reported as an essential tool to catalyse and advance tobacco control, by providing a strong legal framework, political engagement and by accelerating conformity to international standards.
	National and regional coordination and collaboration can support countries in the policy process. Such collaboration should not include tobacco industry, as outlined in article 5.3 of FCTC. Involvement of all relevant ministries and organizations is essential.
Diet	Indicators, standards, and accountability mechanisms (including conflict of interest frameworks, guidelines on engagement, monitoring for self-regulations, and voluntary measures) are crucial for policy success. Strong government leadership, multisectoral and stakeholder engagement are necessary conditions for strengthening the performance of voluntary or non-statutory food reformulation initiatives.
	Knowledge on trade rules is required to implement policies on front of package labelling. Policies need to be implemented within the trade rules and need to demonstrate that policies are the least trade-restricting measure; multinational companies have a great influence because of their economic power, government lobbying and communication and marketing resources. Trade agreements might reduce the scope for governments to implement innovative measures (that have only limited evidence for their effectiveness).
	Limited local evidence and data, lack of research uptake of study findings, no data on food content, consumption, and labelling, perception of weak evidence for the link between diet (eg, SSBs, trans fatty acids, etc) and NCDs, lack of local evidence on interventions effect (eg, SSB) do not support politicians' commitments to agenda setting and policy development. A solid body of evidence is essential to assess the impact of various measures and recommended actions, including evidence on health diets definitions, health and risk distributions among populations, environmental and social values.
	Social acceptability of alcohol and culture around it do shape use and prevalence of social norms. In fact, public opposition and competing interests can interfere with policy processes. Consumers can oppose policy, especially on pricing and shortened licensing hours. Lack of coherence in messaging around policy and appropriate framing of the problem to create political, social, and moral understanding in line with public beliefs and attitudes can help the policy process. Media, as well as civil society play a role in supporting such effort. Community engagement and multisectoral collaboration enable not only better support and accountability.
Physical activity	Physical activity in many countries has received little political attention. In contexts plagued by ongoing conflicts and instability, emerging and recurring epidemics, making the case for physical activity becomes difficult. This requires strong political will and robust scientific evidence of its health benefits. In many LMICs there is still a lack of country and context specific research on physical activity and health, which could be another reason for lower interest of policy-makers to support the promotion of physical activity.
Health system	Multiple components need to be considered to reach effective implementation of clinical guidelines, including assessment of the national protocols and adaptation of WHO PEN protocols to the national context, collection of base line indicators, training of staff in pilot primary healthcare units, implementation of interventions and provision of technical support.
	The use of local data such as STEPS support prioritization of NCDs for public health intervention; NCDs investment case or any other baseline situation assessments can support policy decisions.
Generic	Technical support from global (eg, WHO) and regional organizations and collaborations has been proven to be a key facilitator for knowledge sharing for NCD policy development and primary care.
	A fragmented governance and the absence of a dedicated structure, with a designated body to oversee planning, guidance, monitoring and evaluation of implementation has been a barrier to effective implementation; involvement from multiple actors without adequate coordination by the MoH created silos and fragmentation in policy and program implementation. A strong governance system that facilitates multisectoral collaboration and partnership building is therefore a prerequisite for any NCD policy process.
	Change perception of problem and solution (eg, personal responsibility of risk factors) by using the media to increase public visibility of the issue. Increase the public support for the policy (eg, by organizing a media campaign).
	Define clear accountability frameworks to manage engagement with stakeholders (particularly with private sectors). Change decision-making processes to prevent some opponents from participating. Map the stakeholders: persuade opponents, seek common goals with supporters and expand their participation.

Abbreviations: FCTC, Framework Convention on Tobacco Control; NCD, non-communicable disease; WHO, World Health Organization; PEN, Package of essential noncommunicable; SSB, sugar-sweetened beverage; LMICs, low- and middle-income countries; STEPS, STEPwise approach to surveillance; MoH, Ministry of Health.

**Table 2:** Main Barriers and Facilitators Across Policies

	**Diet**	**Alcohol**	**Tobacco**	**Physical Activity**	**Health System **
**Main Barriers**					
Trade regulations prohibit protective action of policies	x	x	x		
Self-regulatory measures lack transparency and accountability mechanisms		x	x	x	x
Lobbying/influence of industry interfere with policy process	x	x	x	x	
NCDs are framed as individual responsibility and government intervention as patronising (ie, “nanny state”)	x	x	x	x	
Cultural norms impact political decisions	x	x	x	x	x
NCDs lack political and economic support	x	x	x	x	x
Policy making lack of transparency in multisectoral collaboration	x	x	x	x	
Infrastructure is inappropriate (build environment, conflict, and safety)				x	x
Human resources have limited capacity and skills					x
Risk factors are poorly documented and lack data to inform policy	x	x	x	x	x
**Main Facilitators**					
Strong governance and leadership enact legislations and engage in multisectoral action	x	x	x	x	x
Governments manage conflict of interests while engaging with industry	x	x	x	x	
Public awareness, agency, and general health literacy improve the policy process	x	x	x	x	x
Whole-of-society approach support comprehensive action	x	x		x	
Political accountability via social participation and community engagement can improve policy outcomes	x	x	x	x	x
Policy evaluation and monitoring	x	x	x	x	x
International framework with binding powers, such as FCTC, are powerful tools to help governments			x		
Technical support (also via regional coordination) help staff to adopt and implement policies	x	x	x	x	x
Surveillance system and disease specific registries are needed to inform policy makers					x
Local evidence and locally driven policies are better suited for policy development					x

Abbreviations: FCTC, Framework Convention on Tobacco Control; NCD, non-communicable disease.

####  Variable 1: Ability to Define, Adopt a Policy and, When [or “as”] Appropriate, Pass Policy Into Law

 Contextual factors influence the ability to define and pass policies.The adoption (but also implementation) of NCDs policies is markedly shaped by contextual features, such as historical, economic, cultural, and political factors. For instance, globalization, urbanization, adoption of western lifestyles, and geopolitical factors are considered to influence the ability to define and pass NCD policies. African countries, in particular, face competing emergencies, such as a double or triple burden of disease (ie, communicable, non-communicable, injuries) and priorities (ie, *universal health coverage*), which creates tensions in budget allocation and strategic prioritization and planning (Systematic Review [SR] 1, 4, 6, 9, 13, 18, 23, 29, 62, 67, 76, 95, 151). Social and cultural norms and acceptability of unhealthy behaviour and health-seeking behaviours shape use of tobacco, alcohol and food (SR 2, 3, 6, 13, 14, 17). In contexts where specific industries, such as sugar in South Africa, are deeply entrenched (due to historical legacies such as colonialism) and make up a significant part of the economy, it is more difficult to implement diet related “best buys” (SR 42, 47, 110, 114). Similarly for tobacco, countries that are tobacco growers, reported to have limited effectiveness in both formulating and implementing “best buys” interventions related to tobacco control due to economic and political interests (SR 74, 125, 127, 129, 130). In contrast, contexts with national health systems and where there is strong social solidarity favour successful implementation (SR 56). At the health system level, task shifting and training community-based workers for screening and triage, integration of cancer screening into primary care and infectious disease clinics, and use of existing NCD programmes and maternal and child health services for education about primary and secondary cancer prevention (SR 14) were reported as facilitators for defining, adopting and implementing the policies. In relation to physical activity, the built environment shaped the ability to implement physical activity policies, particularly in relation to the ability of communities to adopt less sedentary behaviours (SR 94). Studies from the Caribbean and Nigeria, for instance, reported that physical activity has not been prioritised, since it is often not perceived as important in tackling NCDs (SR 91, 95).

 Several factors can help to explain why policies change, but those related to institutions, interests, ideas, and networks are particularly useful and relevant in the context of best buys policies.^28^

 With regards to the commercial determinants of health (CDoH) *( ie, tobacco, diet, *and* alcohol)*, industry representatives and media were reported as the main actors involved in a way that can hamper or delay both adoption and implementation (SR 3, 5, 7, 29, 30, 33, 42, 101). Industry involvement and lobbying influence how interventions are framed. Risk factors such as unhealthy diet, smoking and alcohol drinking are being consistently framed as an individual responsibility (SR 48, 51), compromising support for population-level policies (SR 8, 10). Consequently, governments that attempt to promote such policies are labelled as a ‘nanny state’ (SR 66, 83, 66).

 With regards to diet, several studies reported ways in which industry lobbies governments and seek to pre-empt enforceable standards (SR 71, 72) by pushing self-regulatory codes (SR 26, 27, 29, 30, 31, 36, 40, 41, 67, 100, 101). For tobacco, industry disseminates ideas that tobacco regulations would not work, would increase illicit trade, create problems for retailers, impact the economy negatively (and livelihood of tobacco farmers), and violate domestic laws and international treaties on IP and investments (SR 117, 133, 137, 142, 145). Consequently, mistrust increases and competing viewpoints fuel political incoherence; neoliberal ideas are often not explicated or interrogated when it comes to efforts to establish policy coherence (SR 146). Additionally, civil society, which could play a key role in advocating for stronger political commitment, is reported not to be present at key decision-making venues (SR 35, 43, 45, 51).

 Where governments are able to frame NCD policies in relation to the high economic costs incurred by secondary and tertiary care, implementation is improved. Food measures such as labelling can be framed as part of a comprehensive policy response, minimizing risk of being contested in trade challenges; a human rights approach to problem framing (SR 12, 72) should be adopted and reframe NCDs using the language of rights, to add weight to health messages and policy reform (SR 24, 26, 31, 32, 40, 41, 100, 111, 113).

 On the other hand, actors that play a facilitating role were bilateral and multilateral agencies (such as UN agencies), philanthropic organizations, regional development banks, academic networks, all played a role in providing legal assistance. WHO in particular has an important role in promoting and monitoring global action against NCDs (SR 12, 71, 73, 75). Policy-makers should establish a platform for meaningful engagement with community members and civil society (SR 2, 5, 6, 8, 9, 10) and multistakeholder engagement and collaboration (SR 115, 118, 123, 134, 136, 141, 145). Research centres within countries provided evidence to Ministry of Health (MoH) and regional organizations (such as Caribbean Community) and support for its members (SR 83).

 The intersection of power and legal capacity related to the CDoH are consistently reported as important barriers. Public health interests may be tempered by participations’ power imbalances, such as Codex or World Trade Organization (WTO) where trade agreements are discussed. Complainants within the trade rules need to demonstrate that policies are the least trade-restricting measure (SR 37, 43, 44, 45, 46). The trade agreement might reduce the scope for governments to implement innovative measures that have only indicative evidence for their effectiveness, due to the regulatory chill effect of the cost of both evidence gathering and defending contested policy (SR 43, 44, 51, 52). For diet-related policies, studies report that industry interferes with the implementation of labelling by using legal strategies to oppose public policies, lobbing for policy substitution, opposing marketing restrictions, advocating against health legislations, using threats and intimidation to discourage approval of international guidelines (SR 27, 29, 31, 33, 38, 54, 104, 110, 114). The action of industry and lobby distort the public health agenda and create competing interests at the policy level (SR 8, 10). NCD policies often challenged at WTO and particularly LMIC face pressures to design regulations in line with WTO (SR 57). For instance, tobacco companies are reported that have raised trade challenges and litigations against tobacco control laws in countries, particularly those without financial means (SR 72, 121, 136, 143).

 On the other hand, examples of opportunities reported by some studies include the use by governments of exemptions to trade agreements (eg, government procurement of local produce); specific WTO agreements could be used for food subsidies used as part of an obesity prevention strategy (SR 43, 44). Additionally, WHO has regulatory and treaty-making powers, enabling it to develop legally binding global conventions, in addition to more common, non-binding World Health Assembly resolutions (such as endorsement of the NCD Global Action Plan) (SR 43); The role of public health researchers and evidence‐based civil society advocacy and strong political will has been key to passing legislation; increased “freedom of speech” and “civil society voices” so that local populations could agitate for top-down change with respect to nutrition and population health challenges; influential actors within communities can play a role in inciting bottom-up awareness (SR 30, 31, 41, 109); the power of civil society and other actors is considered limited but still provided advisers’ contributions for policy development (SR 91).

####  Variable 2: Ability to Implement the Policy

 Sharing expertise, training, and good practices is a key factor in facilitating policy diffusion by creating opportunities to share practical experience in implementing, and enforcing laws and fiscal policies (SR 12, 71, 72, 73). For food policies, a focused advocacy coalition including researchers, civil society health officials, and donors could foster coordinated public health input into Codex processes regarding front-of-package nutrition labelling (SR 35, 46, 51, 37). Technical support (particularly from WHO and experts) has been provided for the development of policies (35, 51, 46) and specific training centres were set up (SR 81, 86). Education of implementation staff on pre-emption practices and trade policies can increase understanding and competencies (SR 24, 32, 33, 36, 38, 100). Pro-active engagement with trade policy makers at early stage of design can help to identify WTO compliance (SR 27, 29, 30, 36, 40, 41, 49, 54, 101, 102, 105, 107).

 Specific capacity and skills to implement policies are required for both implementers and populations. For instance, with regards to *food policies*, charts and labelling require a high level of knowledge and capacity (for implementers) and literacy and agency (for users). Countries faced challenges in developing definitions for a lack of data and guidelines and due to the complexity and variety of market products; there are several difficulties for implementing actors to interpret policies and design campaigns for the population (SR 24, 26, 30, 33, 38, 97, 99, 105). Governments often have limited resources and expertise around Codex issues compared to industry; many countries, particularly in LMIC, lack the knowledge to assess and evaluate healthier replacements (SR 37, 43, 51). Tax increases on *tobacco, *despite being considered relatively politically easy to adopt,seemed to also be difficult “best buys” interventions to implement (eg, keeping tax increases consistent with inflation) (SR 123, 136). The role of legal assistance is poorly understood and highly needed (SR 71). For clinical guidelines, generally, there is a lack of chronic disease prevention training and evidence-based chronic disease programmes and countries lack the capacity to adapt guidelines to local contexts (SR 80).

 A governance system facilitating multisectoral collaboration, partnership building, community mobilisation and social participation, as well as strong leadership and stewardship and coordinate regional action across departments and sectors (SR 12, 68, 71, 73, 75) was seen as a facilitating factor. Such governance is characterised by transparency in both decision making and clear management of conflicts of interest. Policy coherence and accountability of all stakeholders is essential to respond to NCDs challenges. Private sector should comply with regulatory codes and public-private partnerships need to be set up in a transparent manner (SR 12, 56, 57, 72, 75). Countries require improved governance, political leadership, and a whole-of-government approach to making legislative decisions and strengthening regulatory capacities (SR 82). Following the example of Framework Convention on Tobacco Control (FCTC) article 5.3, mechanisms should be developed to protect policies from vested industry (SR 67, 82). The literature reported examples of longstanding effort to engage, organize and mobilize key stakeholder groups that influenced the legislature, including lobbyists who could build trusting relationships with legislators. The creation of the NCD commission allowed for a ‘whole of society’ collaborative approach by including the perspective of civil society and non-health public sectors (SR 91, 92, 96).

 As regards financial capacity, studies (predominantly, but not limited to LMIC) report that countries have limited financial resources (SR 51, 71, 75) and NCD programmes receive insufficient funding within MoH (SR 60, 61, 82). There is limited investment in population health and no funding dedicated to policy adoption and implementation, with overreliance on private and industry finance (SR 31, 33, 53, 105, 106), impacting the financial sustainability of programmes, particularly in LMIC (SR 8, 10). Other programmes, such as HIV focused, tented to receive external funding from donors, while NCD from the national government (SR 83). Hypothecated ‘health’ taxes can help to support NCD efforts and financial mechanisms of reward can help to sustain multisectoral collaboration (SR 12, 56, 68, 75), and earmarked taxes can result in better sustainability (SR 61, 69, 86). Tobacco producing LMIC need to address alternative livelihoods to tobacco production and transition to a more sustainable economy (SR 142).

####  Variable 3: Ability to Monitor Progress, Evaluate and Correct the Course

 Some studies reported a need for better governance principles in managing multistakeholder plans and conflict of interest (SR 71, 75). Where governance is fragmented and resulted in the absence of a dedicated structure, there was no multisectoral action and often work happened in silos (SR 55, 60, 61, 65, 69, 82, 83, 84, 85). Studies reported a general lack of regulatory capacity and procedures for disclosing interactions between governments and industry interference with government policies (SR 63, 69, 82, 85) and lack of transparency on private sector infiltration into policy decisions and financial support of stakeholders (SR 123, 124, 143, 145). Inadequate frameworks and international guidelines for multisectoral collaboration leave ambiguity on how coordination across sectors should be achieved (SR 94) and how to govern conflict of interest and private sector engagement (SR 119, 123, 145). Also, quasi-regulatory or voluntary approaches are compromised by weak standards, targets and commitments, and lack of enforcement mechanisms and monitoring systems.

 Monitoring and evaluation systems are absent or inadequate. The lack of global standardized detail reporting on alcohol control, salt and fat intake, tobacco consumption, etc, hampers countries from monitoring and advancing the NCD control agenda; despite the well-established monitoring and evaluation system of the WHO FCTC, data on expenditure for tobacco control is not routinely updated for many countries (SR 82); the absence of standards or targets make goals hard to achieve and there is limited data on food composition and ingredients’ levels; there is no independent monitoring system and progress reports are not comprehensive or systematic (SR 10, 33, 40, 49, 54, 97, 99, 101, 104, 106); there is a need for a more rigorous method of evaluating policy strength, comprehensiveness and implementation effectiveness (SR 118, 141, 145). Without specifying a magnitude of change and a time frame for achievement, countries cannot evaluate the success or failure of their national policy and actions (SR 2, 93, 96).

 The development of international standards can provide protection from challenges under Technical Barriers to Trade or WTO agreements (SR 35, 44, 45). Studies recommend that if countries decide to adopt self-regulation for nutrition policies, this will require independent monitoring and evaluation of defined and quantifiable targets; monitoring of the food supply and data on trends in health outcomes is needed to inform outcome evaluation (SR 24, 26, 29, 30, 36, 54, 102, 104, 107). Regulatory frameworks acted as enablers for national policy implementation and helped to establish national prioritization and support for countries to establish trade limitations (SR 2, 3, 4, 5, 6, 9). Countries (in all income groups) with the most successful tobacco control policies also have the most active programmes of industry monitoring; (SR 115, 143).

 Availability of local evidence and a deeper understanding of contextual features, as well as mechanisms to measure impact of policies are crucial for effective policy planning (SR 8, 10). Data on food content, consumption and labelling is lacking and difficult to obtain (SR 33, 38, 40, 42, 43, 106, 26, 29, 30, 31, 40, 49, 50, 54, 98, 100, 101, 104). Studies reported the lack of cancer or screening registry, absence of health information systems allowing data linkages; screening programmes are not adapted to local context (SR 13). Where vital registration is not available countries should establish alternative methods such as verbal autopsy as an interim measure, pending improvements to their vital registration system (SR 75); Surveillance systems to monitor NCD risk factors and disease trends (eg, WHO STEPwise approach to surveillance [STEPS]) are necessary to raise awareness and reinforce political commitments for stronger and coordinated multisectoral actions. Technical evidence, such as WHO MPOWER package, supported the development of legislation (SR 67, 69, 86). Scientific publications from research and academic institutions were considered important facilitating factors (SR 117, 118, 119, 121, 131, 136, 145), however, it is not uncommon that industry commissioned reports, surveys, and other forms of evidence; (SR 123, 137, 143, 145). The majority of policies and evidence come from high-income countries and need to be adapted to LMIC (SR 80). Surveillance of the social determinants of health poses challenges due to its scope extending beyond the health sector’s jurisdiction. (SR 81, 86).

###  Suggested Hypothesis: Fragility as a Determinant of NCD Adoption and Implementation

 Studies focusing on fragile settings state several context-specific challenges. A policy disconnect often exists between the burden of disease and national policy responses; in particular, undernutrition is often still considered the focus of policies, with less attention paid to diet as risk factor for NCD development. It is recognised that diet policies should be developed in close association with other related policies in the country, based on cultural considerations, and in collaboration with other sectors; however, the lack of financial support to conduct consultative meetings can represent a barrier to holding and establishing collaborations. Geographical isolation may also contribute to overall political fragility (SR 122, 126, 127, 132). Fragmented health systems, with a mix of private and public health provision are considered complex environment to develop a national plan (SR 20); strengthening public health sector and a political commitment to tackle poverty were seen as contextual enablers (SR 20). Conversely, for countries affected by high refugee influx, humanitarian crises were viewed as windows of opportunity which triggered the activation of action plans to tackle diet within NCD plans (SR 55).

###  Quality Appraisal 

 The quality of the appraised evidence has been evaluated for i) the overall studies; and ii) a subset of findings considered particularly relevant for policy recommendations. Results of the quality appraisal are presented in Supplementary file 4 (Tables S3 and S4) for the individual studies (MMAT) and individual scores of study findings (CERQual), respectively. Of 157 studies included, using MMAT, we identified 93% to be high quality, 6% moderate, and 1% poor. Overall, we consider the body of evidence to be of high quality.When using CERQual, we found similar results for the subset of assessed findings. However, we caution that the findings are highly context-dependent and relevance may be impacted while transferring these results to other contexts.

## Discussion

 This study aimed to systematically review the available global literature on barriers and opportunities of NCD “best buys” and analyse who are the actors, interests and institutions that influence the policy process, in line with Reich PEA framework.^29^ We used a political economy framework to analyse the role of actors and their ideas, mechanisms of engagement, power and finance, and the context in shaping the NCD policy process. We also developed a programme theory based on system thinking that served as our theoretical framework to analyse our findings.

 A recent analysis by Isaranuwatchai et al on ways to support governments to ensure the success of NCD policies,^30^ includes the support to stimulate multisectoral coordination, collaboration and action and introduce systematic thinking. In line with the latter, we have introduced a new way to look at NCD policies by adopting a complexity perspective and using system thinking, in line with recent calls to use complexity theories and methods applied to complex public health interventions.^18,31^ NCD policies are complex interventions that require a system approach that should be integrated in the process of adoption and implementation at a local level. However, implementation research and theory for NCDs are fields that require further expansion, including the use of system methods integrated into the implementation research.^14,32^ With regards to the former recommendation made by Isaranuwatchai et al, our analysis found that multisectoral collaboration is indeed an essential facilitator. It is well known that health in all policies or whole of government approaches are pivotal for the success of public and personal health care policies. Examples of successful multisectoral collaborations as well as technical guidance are available and could support countries to guide dialogues across sectors. Efforts across sectors are essential but clear governance and accountability mechanisms are required, especially concerning the role of private sector. When embarking on the implementation of multisectoral collaboration, it becomes crucial to thoroughly consider and analyze the power dynamics among the involved actors, along with the sources and instruments of power they possess, while also devising effective strategies to manage it. Conducting a power analysis can prove invaluable in unravelling and understanding these power structures, ultimately leading to the formulation of appropriate and effective approaches.^33^

 Greer et al propose the “Health for All Policies” approach as an alternative to the traditional “Health for All Policies” approach.^34^ In HfAP, health is prioritised, and the health sector actively collaborates with other sectors, benefiting both health and other domains simultaneously. It encourages the health sector to take a proactive role in promoting collaboration and achieving shared goals across different sectors.

 An important challenge that emerged strongly from the global literature relates to the engagement of the private sector, especially for population-level policies. Studies report the importance of reinforcement of accountability and transparency mechanisms, achievable via implementation of strict eligibility criteria for joining partnerships, the adoption of ethical codes of conduct among stakeholders involved in policy formulation, and publicly available information regarding processes and industry submissions to consultations. Firstly, the involvement of private sector is not fully governed by clear guidelines, which open the way to conflict of interests and the influence of powerful industries. The so-called CDoH^35^ have been increasingly studied, but progress to manage them are lacking. In fact, while a clear framework exists to govern the involvement of the tobacco industry in policy negotiations such as FCTC, clear guidelines for alcohol and diet are far to be satisfactory (eg, Framework of Engagement with Non-State Actors^36^). Some have suggested a human right-based approach^37^ or making the case of using WTO exemptions or other strategies^38^ to overcome some of these challenges. As trade rules represent a key challenge for governments to implement NCD policies, the impact of trade rules on NCD prevention needs to be assessed during trade negotiations. Policy-makers must take measures to ensure that trade rules support, rather than hinder, efforts to prevent and control NCDs. This can be achieved by supporting greater strategic and informed engagement between the health and trade policy sectors and ensuring a high level of health protection in trade and investment agreements with cooperation between disciplines, engagement with experts in law, economics and public health policy, and fully transparent policy processes and governance structures.^39^

 How health policy debates are framed also plays an important role. The “framing” of risk factors by different actors (private and public stakeholders) shapes the agenda-setting process to determine which policies will receive political attention and how. Private sector tended to frame diet or physical activity as personal responsibility influencing political perception and prioritization. Framing also influences support (or opposition) that group(s) of people can provide to policy. For instance, tobacco farmers change in attitude in Kenya towards industry was a prerequisite to advancement in the tobacco control.^40^ Framing analysis can help to identify these challenges or opportunities and define clear interventions that can sustain health policies. An improved engagement of civil society on NCD matters can support the prioritization in the national and international policy agenda and their correct framing.^41^

 Finally, contextualisation is essential.^30^ Isaranuwatchai et al called “contested buys” those best buys that lack local cost-effectiveness data – and for a reason. Several studies reported that the absence of locally informed strategies hamper the implementation of best buys. While the overarching strategies and recommendations for action may have universal validity, policies tailored on local knowledge and data are required. Particularly, fragile states often lack resources and epidemiological data to establish locally defined policies. Additionally, fragile settings may also face the biggest burden of disease and lack of resources, requiring global solidarity to build sustainable solutions (away from donor dependency).

 Health services such as screening or drug therapies also present numerous challenges, despite being the most widely implemented policies. Challenges include, for instance, the incorporation of NCD services into mixed health systems (ie, systems with public and private components) and into services that have been till today directed towards infectious diseases. Task shifting and integration of NCD and communicable disease services should be adopted when possible. In this sense, recent lessons on the learning^42^ and strengthening health systems^43^ can be applied to NCD policy to develop effective strategies.

 Overall, we know which policies and intervention work and need to be implemented; however, we do not necessarily know how to adapt and implement these policies. Implementation research is essential to understand what policies should be adopted, ensure that they will be implemented as planned and contextualised, and integrated into *universal health coverage*, health system strengthening and comprehensive PHC approaches. Implementation research can help to close the gap between evidence and practice, tailoring interventions to local contexts, building a robust evidence base and optimizing resources. NCD Strategies and Plans are complex, and their implementation requires a thorough analysis of the whole system during initial planning to ensure robust implementation and overcome the barriers that countries are currently facing.^44-46^

 This review article identifies critical gaps in the existing literature on NCD implementation and highlights key focus areas for future research. The study emphasizes the significance of implementation research for NCDs, the need to analyze diverse actors’ roles beyond industry, and to comprehend dynamics across various sectors. Furthermore, it underscores the importance of learning from multiple unhealthy industries, considering their unique characteristics.^12^ A deeper exploration of the drivers that influence political decisions, beyond the oversimplification of “political will,” such as neoliberalism’s impact on political decisions,^47^ or the desire to win or stay in government,^48^ is also recommended. Future research should explore the advancement of complexity methods to support NCDs policy processes and explicitly unravel power dynamics, especially concerning the CDoH. Understanding the long-term effectiveness and sustainability of different interventions, assessing challenges and opportunities for primary healthcare integration, analyzing the economic impact of NCDs, and evaluating intervention cost-effectiveness in LMIC and potential health-economic trade-offs are crucial research imperatives. Additionally, fostering interdisciplinary research that unites experts from diverse fields is vital for a comprehensive understanding of NCD implementation barriers and developing effective health system strengthening strategies.

###  Strengths and Limitations

 As far as we are aware, this is the first review that aimed to systematically assess the barriers and opportunities to the adoption and implementation of NCD “best buys.” We adopted a complexity systematic review approach with the development of a programme theory to show how system thinking can be beneficial in studying health policy and complementing implementation research and theory. Despite this study’s methodological and theoretical rigour, there are important limitations to note. Firstly, the research team developed the programme theory without consultation with other external stakeholders. We are planning to refine this model in a follow-up project with key stakeholders at a global level, which may find different dynamics and highlight additional feedback loops. Secondly, we acknowledge that this study may not be comprehensive since we decided to limit our focus on studies that used a political economy lens. We retrieved limited information for some of the best buys (eg, physical activity) and this may affect the generalisability of findings. Thirdly, our search resulted in a large number of results and heterogeneity in included studies. Double screening of articles and double extraction of data was not practical given this volume of material. We mitigated the risk of bias by a double screening of a random 20% of search results and data extraction sheets from all reviewers being checked for quality and consistency by a second researcher. However, given the main studies retrieved were qualitative and that our synthesis approach was narrative, we note that analyses indicated a saturation of the concepts under study, and we consider that any missing literature is unlikely to significantly alter findings.

## Conclusions

 Studying the political economy of NCD forced us to see how the distribution of power and resources largely impact the progress of NCD and how such factors are shared across low-, middle-, and high-income countries. To address this global challenge effectively, nations must strengthen capabilities at the institutional level, prioritise knowledge exchange, equitable resource allocation, global collaboration, and swift action on the CDoH. Implementation research with a political economy perspective can help to contextualise and implement interventions effectively. Using tools like power analysis, systems thinking, and embedded implementation research is key for researchers and policy-makers to advance NCD prevention and control.

## Ethical issues

 The study used secondary data and did not require ethical approval.

## Competing interests

 Authors declare that they have no competing interests.

## Funding

 This study was funded by NIHR Global Health Research Programme (16/136/100). The views expressed are those of the authors and not necessarily those of the National Health Service, the NIHR or the Department of Health.

## Supplementary files


Supplementary file 1. Search Strategy.

Supplementary file 2 contains Table S1.

Supplementary file 3 contains Table S2.

Supplementary file 4 contains Tables S3 and S4.
