# Supplementary material for: Barriers and Opportunities for WHO ‘Best Buys’ Non-Communicable Disease Policy Adoption and Implementation From a Political Economy Perspective: A Complexity Systematic Review
Source: Int J Health Policy Manag. 2024 Feb 4;13:7989. doi: 10.34172/ijhpm.2023.7989 (PMC11016278; doi:10.34172/ijhpm.2023.7989)
Supplement: Supplementary file 1 — Search Strategy. [file ijhpm-13-7989-s001.pdf]

Article title: Barriers and Opportunities for WHO "Best Buys" Non-communicable Disease Policy Adoption and Implementation From a Political Economy Perspective: A Complexity Systematic Review

Journal name: International Journal of Health Policy and Management (IJHPM)

Authors' information: Giulia Loffreda[1]*, Stella Arakelyan[2,1], Ibrahim Bou-Orm[1], Hampus Holmer[3], Luke N. Allen[4], Sophie Witter[1], Alastair Ager[1], Karin Diaconu[1]

[1]NIHR Research Unit of Health in Fragility, Institute for Global Health and Development, Queen Margaret University Edinburgh, Musselburgh, UK.

[2]Advanced Care Research Centre, Usher Institute, University of Edinburgh, Edinburgh, UK.

[3]Department of Global Public Health, Karolinska Institutet, Stockholm, Sweden.

[4]Department of Clinical Research, London School of Hygiene and Tropical Medicine, London, UK.

***Correspondence to:** Giulia Loffreda; Email: gloffreda@qmu.ac.uk

Citation: Loffreda G, Arakelyan S, Bou-Orm I, et al. Barriers and opportunities for WHO "best buys" non-communicable disease policy adoption and implementation from a political economy perspective: a complexity systematic review. Int J Health Policy Manag. 2024;13:7989. doi:10.34172/ijhpm.2023.7989

Supplementary file 1. Search strategy

A. Search Strategy/Search Terms (MEDLINE)

1. cardiovascular diseases/ or heart diseases/ or vascular diseases/ or cerebrovascular diseases/
2. exp Myocardial Ischemia/
3. Heart Failure/
4. exp brain ischemia/ or exp stroke/
5. exp Diabetes Mellitus, Type 2/
6. lung diseases, obstructive/ or exp pulmonary disease, chronic obstructive/
7. exp *Neoplasms/
8. ((cardiovascular or cardio-vascular) adj3 disease*).ti,ab.
9. ((cardiovascular or cardio-vascular) adj3 (event* or outcome* or risk*)).ti,ab.
10. ((coronary or heart or myocard*) adj3 disease*).ti,ab.
11. ((coronary or heart or myocard*) adj3 (event* or outcome* or risk*)).ti,ab.
12. ((ischaemic or ischemic or ischaemia or ischemia) adj3 disease*).ti,ab.
13. ((ischaemic or ischemic or ischaemia or ischemia) adj3 (event* or outcome* or risk*)).ti,ab
14. myocardial infarct*.ti,ab.

15. ((cerebrovascular or vascular) adj3 disease*).ti,ab.
16. ((cerebrovascular or vascular) adj3 (event* or outcome* or risk*)).ti,ab.
17. stroke.ti,ab.
18. heart failure.ti,ab.
19. diabet*.ti.
20. ((type 2 or type ii or noninsulin dependent or non insulin dependent or adult onset or maturity onset or obes*) adj2 diabet*).ti,ab.
21. (niddm or t2dm or tiidm).ti,ab.
22. (chronic adj2 (lung or pulmonary)).ti,ab.
23. chronic obstructive pulmonary disease.ti,ab.
24. (neoplas* or cancer* or carcinoma* or tumor* or tumour* or malignan* or leukaemia or leukemia or lymphoma?).ti,ab.
25. 1 or 2 or 3 or 4 or 5 or 6 or 7 or 8 or 9 or 10 or 12 or 13 or 14 or 15 or 16 or 17 or 18 or 19 or 20 or 21 or 22 or 23 or 24
26. Taxes/ and ("Tobacco Use"/ or exp "Tobacco Use Cessation"/ or drinking behavior/ or exp alcohol drinking/ or exp dietary fats/ or Sodium Chloride/)
27. (tax or taxes or taxing or taxation).ti.
28. ((food? or diet* or vegetable? or fruit? or sugar* or fat or fats or sucrose or candy or sweet* or snack* or fastfood? or junkfood?) and (tax or taxes or taxing or taxation)).ti,ab.
29. ((smok* or tobacco) and (tax or taxes or taxing or taxation)).ti,ab.
30. ((alcohol or drinking) and (tax or taxes or taxing or taxation)).ti,ab.
31. ((smok* or tobacco) and (subsidy or subsidies or incentiv* or voucher?)).ti,ab.
32. ((alcohol or drinking) and (subsidy or subsidies or incentiv* or voucher?)).ti,ab.
33. (Social Control, Formal/ or Legislation/) and ("Tobacco Use"/ or exp "Tobacco Use Cessation"/ or Tobacco Industry/ or Tobacco Smoke Pollution/ or drinking behavior/ or exp alcohol drinking/ or exp dietary fats/ or fast foods/ or Sodium Chloride/)
34. smoke-free policy/
35. exp Nutrition Policy/
36. ((smoke or smoking or tobacco) adj2 (ban or bans or banned or free)).ti,ab.
37. ((smoke or smoking or tobacco) and (legislat* or law? or regulation or regulatory)).ti,ab.
38. ((alcohol or drinking) and (legislat* or law? or regulation or regulatory)).ti,ab.
39. exp Trans Fatty Acids/
40. Social Marketing/
41. (Marketing/ or advertising as topic/ or Mass Media/ or product packaging/ or product labeling/) and ("Tobacco Use"/ or exp "Tobacco Use Cessation"/ or drinking behavior/ or exp

alcohol drinking/ or exp Diet/ or food/ or exp dietary fats/ or fast foods/ or fruit/ or vegetables/ or Sodium Chloride, Dietary/ or Beverages/ or exp Exercise/ or Motor Activity/ or health behavior/ or risk reduction behavior/)

42. food packaging/ or food labeling/
43. ((food? or diet* or vegetable? or fruit? or sugar* or fat or fats or sucrose or candy or sweet* or snack* or fastfood? or junkfood?) and (marketing or adverti?ing or sponsorship? or label* or pack*)).ti,ab.
44. ((smok* or tobacco) and (marketing or adverti?ing or sponsorship? or label* or pack*)).ti,ab.
45. exp alcohol drinking/ and retail.mp
46. exp alcohol drinking/ and restriction.mp
47. ((alcohol drinking) and (marketing or adverti?ing or sponsorship? or label* or pack*)).ti,ab.
48. (salt adj3 (intake or reduc* or lower*)).ti,ab.
49. Health Promotion/ and (Neoplasms/ or Obesity/ or Diabetes Mellitus, Type 2/ or Smoking/ or Risk Factors/ or Cardiovascular Diseases/ or Hypertension/)
50. Drug therapy, combination and (Diabetes Mellitus, Type 2/ or Cardiovascular Diseases/ or Hypertension/)
51. Patient Education as Topic/ or Counseling/ or Patient Compliance/ or Motivational Interviewing/ and (Neoplasms/ or Obesity/ or Diabetes Mellitus, Type 2/ or Smoking/ or Risk Factors/ or Cardiovascular Diseases/ or Hypertension/)
52. Health Education/ or Health Knowledge, Attitudes, Practice/ and (Life Style/ or Food Habits/ or Diet/ or Health Behavior/ or Obesity/ or Motor Activity/
53. Hydroxymethylglutaryl-CoA Reductase Inhibitors/ or Simvastatin/ or Aspirin/ or Metformin/ or Adrenergic beta-Antagonists/ or Propranolol/
54. Food legislation/ and (dietary fats/ or sodium/)
55. Food supply/ and (dietary fats/ or sodium/)
56. Papanicolaou Test/
57. Mass Screening/ and (Uterine Cervical Neoplasms/ or Cervical Intraepithelial Neoplasia/)
58. ((cervical or pap) adj3 screen*).ti,ab.
59. Hepatitis B Vaccines/
60. ((hepatitis b or hep b) adj3 (vaccin* or immuni?ation or immuni?e)).ti,ab.
61. *Aspirin/
62. Primary Prevention/ and Aspirin/
63. (aspirin or acetylsalicylic acid).ti,ab.
64. best Buys.mp
65. or/26-64
66. 25 and 65

B. Search strategy for grey literature

After different attempts to optimise the literature search for the grey literature, we used the following search terms, and relevant variations adapted either to Google Scholar or WHO IRIS.

1. "Non-communicable disease" AND "policy adoption"

2. "Non-communicable disease" AND "policy implementation"

3. "Chronic disease" AND "policy evaluation”
