# Supplementary material for: Barriers and Opportunities for WHO ‘Best Buys’ Non-Communicable Disease Policy Adoption and Implementation From a Political Economy Perspective: A Complexity Systematic Review
Source: Int J Health Policy Manag. 2024 Feb 4;13:7989. doi: 10.34172/ijhpm.2023.7989 (PMC11016278; doi:10.34172/ijhpm.2023.7989)
Supplement: Supplementary file 2 — contains Table S1. [file ijhpm-13-7989-s002.pdf]

Article title: Barriers and Opportunities for WHO "Best Buys" Non-communicable Disease Policy Adoption and Implementation From a Political Economy Perspective: A Complexity Systematic Review

Journal name: International Journal of Health Policy and Management (IJHPM)

Authors' information: Giulia Loffreda[1]*, Stella Arakelyan[2,1], Ibrahim Bou-Orm[1], Hampus Holmer[3], Luke N. Allen[4], Sophie Witter[1], Alastair Ager[1], Karin Diaconu[1]

[1]NIHR Research Unit of Health in Fragility, Institute for Global Health and Development, Queen Margaret University Edinburgh, Musselburgh, UK.

[2]Advanced Care Research Centre, Usher Institute, University of Edinburgh, Edinburgh, UK.

[3]Department of Global Public Health, Karolinska Institutet, Stockholm, Sweden.

[4]Department of Clinical Research, London School of Hygiene and Tropical Medicine, London, UK.

***Correspondence to:** Giulia Loffreda; Email: gloffreda@qmu.ac.uk

Citation: Loffreda G, Arakelyan S, Bou-Orm I, et al. Barriers and opportunities for WHO "best buys" non-communicable disease policy adoption and implementation from a political economy perspective: a complexity systematic review. Int J Health Policy Manag. 2024;13:7989. doi:10.34172/ijhpm.2023.7989

Supplementary file 2

Table S1. Inclusion and Exclusion Criteria for full-text screening of articles.

Inclusion Criteria	Include	Exclude
Population	Any population where NCD policy was developed at national, regional, and international level	
Intervention	NCDs-related defined as:<br>• Consider, the multi or individual factorial drivers of one or multiple NCD/s, and related public health or the social determinants of health; or<br>• Involve a policy or intervention in line with the 16 WHO Best buys, these may involve:<br>• transformative coordinated action (including policies, strategies, practices) across a broad range of disciplines and stakeholders, including partners outside traditional health sectors;<br>• Operate across all levels of governance, including the local level so that such approaches are reinforced and sustained, and<br>• Identify and target opportunities throughout the life course (from infancy to old age)	Studies which looked at only one portion of the population or individual level approach, behavioral change

Outcome	Outcomes are defined as:<br><br>Barriers and/or facilitators to adoption and implementation from a political economy perspective and may include (but not limited to):<br><br>Organizational outcomes e.g., cross-sector collaboration; new partnerships; environmental changes; resource allocation; leadership etc.<br><br>Process outcomes, e.g., what each project aimed to achieve and barriers and facilitating factors associated with achieving or not achieving<br><br>Central level: funding, legal frameworks for implementation, etc. (or lack thereof)<br><br>Decentralized level: management/support skills, access to frontlines, enforcement power, communication/information/understanding (or lack thereof), competing priorities/values/ideas/interests, etc.<br><br>Frontline level: staffing, skills, infrastructure/materials, communication/information/understanding of the policy, competing priorities/values/ideas/interests, Communities/demand side: information/understanding, cultural barriers, financial barriers, access/geographical barriers, etc. Outcomes may be at population level, national/regional/global level.	Cost effectiveness; not reporting outcomes under study.
Study design	Primary/empirical research. These may be randomized controlled trials (RCTs) or non-RCTs, natural experiments, mixed methods studies, policy analysis, key informant interviews; policy documents on NCD prevention (including acts and laws, strategic plans, guidelines and government directives), case studies in successful policy formulation and implementation at national/international level.	Case-control studies; clinical studies; behavioral; etiological; systematic and scoping reviews; literature reviews.
