# Supplementary material for: Barriers and Opportunities for WHO ‘Best Buys’ Non-Communicable Disease Policy Adoption and Implementation From a Political Economy Perspective: A Complexity Systematic Review
Source: Int J Health Policy Manag. 2024 Feb 4;13:7989. doi: 10.34172/ijhpm.2023.7989 (PMC11016278; doi:10.34172/ijhpm.2023.7989)
Supplement: Supplementary file 3 — contains Table S2. [file ijhpm-13-7989-s003.pdf]

Article title: Barriers and Opportunities for WHO "Best Buys" Non-communicable Disease Policy Adoption and Implementation From a Political Economy Perspective: A Complexity Systematic Review

Journal name: International Journal of Health Policy and Management (IJHPM)

Authors' information: Giulia Loffreda[1]*, Stella Arakelyan[2,1], Ibrahim Bou-Orm[1], Hampus Holmer[3], Luke N. Allen[4], Sophie Witter[1], Alastair Ager[1], Karin Diaconu[1]

[1]NIHR Research Unit of Health in Fragility, Institute for Global Health and Development, Queen Margaret University Edinburgh, Musselburgh, UK.

[2]Advanced Care Research Centre, Usher Institute, University of Edinburgh, Edinburgh, UK. [3]Department of Global Public Health, Karolinska Institutet, Stockholm, Sweden.

[4]Department of Clinical Research, London School of Hygiene and Tropical Medicine, London, UK.

***Correspondence to:** Giulia Loffreda; Email: gloffreda@qmu.ac.uk

Citation: Loffreda G, Arakelyan S, Bou-Orm I, et al. Barriers and opportunities for WHO "best buys" non-communicable disease policy adoption and implementation from a political economy perspective: a complexity systematic review. Int J Health Policy Manag. 2024;13:7989. doi:10.34172/ijhpm.2023.7989

Supplementary file 3

Table S2. Characteristics of studies

No.	Policy area	Reference	Country of study	Context classification	Method
1	Alcohol	Abiona, O., Oluwasanu, M. & Oladepo, O. Analysis of alcohol policy in Nigeria: multi-sectoral action and the integration of the WHO "best-buy" interventions. *BMC Public Health* **19,** 810 (2019). https://doi.org/10.1186/s12889-019-7139-9	Nigeria	Fragile	Mixed method
2	Alcohol	Colin Angus, John Holmes, and Petra S Meier. 2019. "Comparing Alcohol Taxation Throughout the European Union." Addiction 114: 1489–1494. https://doi.org/10.1111/add.14631.	28 EU Member	Non-fragile (HIC + MIC)	Descriptive analysis
3	Alcohol	David A. Keatley, Sarah J. Hardcastle, Natacha Carragher, Tanya N. Chikritzhs, Mike Daube, Adam Lonsdale, and Martin S. Hagger. 2018. "Attitudes and Beliefs Towards Alcohol Minimum Pricing in Western Australia." Health Promotion International 33 (3): 400–409. https://doi.org/10.1093/heapro/daw092	Australia	Non-fragile (HIC)	Policy analysis

4	Alcohol	Mary Lawhon and Clare Herrick. 2013. "Alcohol Control in the News: The Politics of Media Representations of Alcohol Policy in South Africa." *Journal of Health Politics, Policy, and Law* 38 (5): 987–1021. doi: 10.1215/03616878-2334683. Epub 2013 Jun 21. PMID: 23794743.	South Africa	Non-fragile (UMIC)	Document analysis
5	Alcohol	Jessica Li, Melanie Lovatt, Douglas Eadie, Fiona Dobbie, Petra Meier, John Holmes, Gerard Hastings, and Anne Marie MacKintosh. 2017. "Public Attitudes Towards Alcohol Control Policies in Scotland and England: Results from a Mixed-Methods Study." Social Science and Medicine 177: 177–189. https://doi.org/10.1016/j.socscimed.2017.01.037	UK	Non-fragile (HIC)	Mixed method
6	Alcohol	Rupali J. Limaye, Lainie Rutkow, Rajiv N. Rimal, and David H Jernigan. 2014. "Informal Alcohol in Malawi: Stakeholder Perceptions and Policy Recommendations." Journal of Public Health Policy 35: 119–131. https://doi.org/10.1057/jphp.2013.43	Malawi	Non-fragile (LIC)	Qualitative method
7	Alcohol	Beatrice L. Matanje Mwagomba, Misheck J. Nkhata, Alex Baldacchino, Jennifer Wisdom, and Bagrey Ngwira. 2018. "Alcohol Policies in Malawi: Inclusion of WHO "Best Buy" Interventions and Use of Multi-Sectoral Action." BMC Public Health 18: 957. https://doi.org/10.1186/s12889-018-5833-7	Malawi	Non-fragile (LIC)	Case study
8	Alcohol	Eva Jané-Llopis, Daša Kokole, Maria Neufeld, Omer Syed Muhammad Hasan, and Jürgen Rehm. 2020. "What Is the Current Alcohol Labelling Practice in the WHO European Region and What Are Barriers and Facilitators to Development and Implementation of Alcohol Labelling Policy?" Health Evidence Network Synthesis Report No. 68. Copenhagen: WHO Regional Office for Europe.	Europe	Non fragile (HIC)	Case study
9	Alcohol	Kidong Park. 2019. "New Law on Prevention and Control of Alcohol Related Harms in Vietnam." Journal of Global Health Science 1 (2): e49. https://doi.org/10.35500/jghs.2019.1.e49	Vietnam	Non-fragile (LMIC)	Qualitative method
10	Alcohol	Dag Rekve, Nicholas Banatvala, Adam Karpati, Dudley Tarlton, Lucinda Westerman, Kristina Sperkova, Sally Casswel, Maik Duennbie, Ariella Rojhani, Øystein Bakke , Maristela Monteiro, Natalia Linou, Alexey Kulikov, and Vladimir B Poznyak. 2019. "Prioritising Action on Alcohol for Health and Development." *BMJ* 367: l6162. doi:10.1136/bmj.l6162	Global	Global	Policy analysis
11	Alcohol	Karen Wallace and Bayard Roberts. 2014. "An Exploration of the Alcohol Policy Environment in Post-Conflict Countries." Alcohol and Alcoholism 49 (3): 356–362: https://doi.org/10.1093/alcalc/agt142	conflict affected states	Fragile	Case study
12	Alcohol, diet, tobacco,physical activity	Vivian Lin and Bronwyn Carter. 2013. "From Healthy Public Policy to Intersectoral Action and Health-in-All Policies." In Global Handbook on Noncommunicable Diseases and Health Promotion, edited by D. McQueen, 189–201. New York: Springer. https://doi.org/10.1007/978-1-4614-7594-1_12	Global case studies	Global	Mixed method
13	Cancer	Ranajit Mandal and Partha Basu. 2018. "Cancer Screening and Early Diagnosis in Low and Middle Income Countries." Bundesgesundheitsbl 61: 1505–1512. https://doi.org/10.1007/s00103-018-2833-9	LMICs	Global	Document analysis
14	Cancer	Malcolm A. Moore. 2014. "Cancer Control Programs in East Asia: Evidence from the International Literature." Journal of Preventative Medicine and Public Health 47 (4). https://doi.org/10.3961/jpmph.2014.47.4.183	East Asia	Non fragile (HIC+MIC)	Case study
15	Cancer	Justin O. Parkhurst and Madhulika Vulimiri. 2013. "Cervical Cancer and the Global Health Agenda: Insights from Multiple Policy-Analysis Frameworks." Global Public Health 8 (10): 1093–1108. doi: 10.1080/17441692.2013.850524	Global	Global	Policy analysis
16	Cancer	Diana Sarfati, Rachel Dyer, Filipina Amosa-Lei Sam, et al. 2019. "Cancer Control in the Pacific: Big Challenges Facing Small Island States." *Lancet Oncology* 20 (9): e475–e492.	Pacific island countries and territories	Fragile (MIC) [not for OECD]	Case study
17	Cancer	Dingle Spence, Rachel Dyer, Glennis Andall-Brereton, et al 2019. "Cancer Control in the Caribbean Island Countries and Territories: Some Progress but the Journey Continues." *Lancet Oncology* 20 (9): e503–e521.	Carribean Island countries	Non-fragile (MIC)	Case study
18	Cancer	Silvina Frech, Catherine A. Muha, Lisa M. Stevens, et al 2018. "Perspectives on Strengthening Cancer Research and Control in Latin America Through Partnerships and Diplomacy: Experience of the National Cancer Institute's Center for Global Health." *Journal of Global Oncology* 4: 1-11. doi:10.1200/JGO.17.00149	Latin America	Non fragile (MIC)	Case study

19	Diabetes	Willemijn E. De Bruin, Cherie Stayner, Michel de Lange, and Rachael W. Taylor. 2018. "Who Are the Key Players Involved with Shaping Public Opinion and Policies on Obesity and Diabetes in New Zealand?" Nutrients 10 (11): 1592. https://doi.org/10.3390/nu10111592	New Zealand	Non-fragile (HIC)	Social Network analysis
20	Diabetes	Tint Swe Latt, Than Than Aye, Ko Ko, and Ko Ko Zaw. 2016. "Gaps and Challenges to Integrating Diabetes Care in Myanmar." WHO South-East Asia Journal of Public Health 5 (1): 48–52. https://doi.org/10.4103/2224-3151.206553	Myanmar	Fragile (LMIC)	Document analysis
21	Diabetes	Linda Penn, Angela Rodrigues, Anna Haste, Marta M Marques, Kirsten Budig, Kirby Sainsbury, Ruth Bell, Vera Araújo-Soares, Martin White, Carolyn Summerbell, Elizabeth Goyder, Alan Brennan, Ashley J Adamson, and Falko F Sniehotta. 2018. "NHS Diabetes Prevention Programme in England: Formative Evaluation of the Programme in Early Phase Implementation." *BMJ Open* 8: e019467. doi: 10.1136/bmjopen-2017-019467	England	Non-fragile (HIC)	Mixed method
22	Diabetes	André M. N. Renzaho. 2015. "The Post-2015 Development Agenda for Diabetes in Sub-Saharan Africa: Challenges and Future Directions." *Global Health Action* 8: 27600. https://doi.org/10.3402/gha.v8.27600	Sub-Saharan Africa	Non-fragile (UMIC)	Case study
23	Diabetes	Veronica Shiroya, Florian Neuhann, Olaf Müller, and Andreas Deckert. 2019. "Challenges in Policy Reforms for Non-Communicable Diseases: The Case of Diabetes in Kenya." Global Health Action 12: 1. doi: 10.1080/16549716.2019.1611243	Kenya	Fragile (LMIC)	Document analysis + interviews
24	Diet	Joao Breda, Lea Samant, Nash Castro, Stephen Whiting, Julianne Williams, Jo Jewell, Kaia Engesveen, and Kremlin Wickramasinghe. 2020. "Towards Better Nutrition in Europe: Evaluating Progress and Defining Future Directions." Food Policy 96: 101887. https://doi.org/10.1016/j.foodpol.2020.101887	WHO European Region	Non-fragile (HIC + MIC)	Survey
25	Diet	Tracy Comans, Nicole Moretto. and Joshua Byrnes. 2017. "Public Preferences for the Use of Taxation and Labelling Policy Measures to Combat Obesity in Young Children in Australia." International Journal of Environmental Research and Public Health 14 (3): 324. https://doi.org/10.3390/ijerph14030324	Australia	Non-fragile (HIC)	Survey
26	Diet	A. Gesser-Edelsburg, R. Endevelt, and Y. Tirosh-Kamienchick. 2014. Nutrition Labelling and the Choices Logo in Israel: Positions and Perceptions of Leading Health Policy Makers. Journal of Human Nutrition and Dietetics: The Official Journal of the British Dietetic Association 27 (1): 58–68. https://doi.org/10.1111/jhn.12050	Israel	Non-fragile (HIC)	Qualitative method
27	Diet	Melissa Mialon, Jonathan Mialon, Giovanna Calixto Andrade, and Moubarac Jean-Claude. 2020. "'We Must Have a Sufficient Level of Profitability': Food Industry Submissions to the French Parliamentary Inquiry on Industrial Food." Critical Public Health 30 (4): 457–467. doi: 10.1080/09581596.2019.1606418	France	Non-fragile (HIC)	Document analysis
28	Diet	Melissa Mialon, Boyd Swinburn, Steven Allender, and Gary Sacks. 2016. "Systematic Examination of Publicly-Available Information Reveals the Diverse and Extensive Corporate Political Activity of the Food Industry in Australia." BMC Public Health 16: 283. https://doi.org/10.1186/s12889-016-2955-7	Australia, UK	Non-fragile (HIC)	Case study
29	Diet	Melissa Mialon, Boyd Swinburn, Jillian Wate, Isimeli Tukana, and Gary Sacks. 2016. "Analysis of the Corporate Political Activity of Major Food Industry Actors in Fiji." Global Health 12: 18. https://doi.org/10.1186/s12992-016-0158-8	Fiji	Non-fragile (UMIC)	Mixed method
30	Diet	R. Pérez-Escamilla, C.K. Lutter, C. Rabadan-Diehl, A. Rubinstein, A. Calvillo, C. Corvalán, C. Batis, E. Jacoby, S. Vorkoper, L. Kline, E. Ewart-Pierce, and J.A. Rivera. 2017. "Prevention of Childhood Obesity and Food Policies in Latin America: From Research to Practice." Obesity Reviews : An Official Journal of the International Association for the Study of Obesity 18 Suppl 2: 28–38. https://doi.org/10.1111/obr.12574	Mexico, Chile, Ecuadaor, Argentina	Non-fragile (UMIC)	Case study
31	Diet	Tarryn Phillips, Amerita Ravuvu, Celia McMichael, Anne Marie Thow, Jennifer Browne, Gade Waqa, Jillian Tutuo, and Deborah Gleeson. 2021. "Nutrition Policy-Making in Fiji: Working in and Around Neoliberalisation in the Global South." Critical Public Health 31 (3): 316–326. doi: 10.1080/09581596.2019.1680805	Fiji	Non-fragile (UMIC)	Mixed method
32	Diet	Jennifer L Pomeranz, Leslie Zellers, Michael Bare, and Mark Pertschuk. 2019. "State Preemption of Food and Nutrition Policies and Litigation: Undermining Government's Role in Public Health." *American Journal of Preventive Medicine* 56 (1): 47–57.	USA	Non-fragile (HIC)	Policy analysis

33	Diet	Erica Reeve, Anne Marie Thow, Colin Bell, Katrin Engelhardt, Ella Cecilia Gamolo-Naliponguit, John Juliard Go, and Gary Sacks. 2018. "Implementation Lessons for School Food Policies and Marketing Restrictions in the Philippines: A Qualitative Policy Analysis." Global Health 14: 8. https://doi.org/10.1186/s12992-017-0320-y	Philippines	Non-fragile (UMIC)	Qualitative method
34	Diet	C. Scott, B. Hawkins, and C. Knai. 2017." Food and Beverage Product Reformulation as a Corporate Political Strategy." Social Science and Medicine 172: 37–45. https://doi.org/10.1016/j.socscimed.2016.11.020	USA	Non fragile (HIC)	Policy analysis
35	Diet	B. Winburn. 2013. "Monitoring and Benchmarking Government Policies and Actions to Improve the Healthiness of Food Environments: A Proposed Government Healthy Food Environment Policy Index." Obesity Reviews: An Official Journal of the International Association for the Study of Obesity 14 Suppl 1: 24–37. https://doi.org/10.1111/obr.12073	Global	Global	Mixed method
36	Diet	Anne Marie Thow, Alexandra Jones, Corinna Hawkes, Iqra Ali, and Ronald Labonté. 2018. "Nutrition Labelling Is a Trade Policy Issue: Lessons from an Analysis of Specific Trade Concerns at the World Trade Organization." Health Promotion International 33 (4): 561–571. https://doi.org/10.1093/heapro/daw109	Chile, Indonesia, Peru, Ecuador. Thailand	Non-fragile (MIC)	Document analysis
37	Diet	Anne Marie Thow Alexandra Jones, Carmen Huckel Schneider, and Ronald Labonté. 2019. "Global Governance of Front-of-Pack Nutrition Labelling: A Qualitative Analysis." Nutrients 11 (2): 268. https://doi.org/10.3390/nu11020268	global	Global	Qualitative method
38	Diet	Gade Waqa, Marj Moodie, Wendy Snowdon, Catherine Latu, Jeremaia Coriakula, Steven Allender, and Colin Bell. 2017. "Exploring the Dynamics of Food-Related Policymaking Processes and Evidence Use in Fiji Using Systems Thinking." Health Research and Policy Systems 15: 74. https://doi.org/10.1186/s12961-017-0240-6	Fiji	Non-fragile (UMIC)	Qualitative method
39	Diet	Adrián Alberto Díaz, Paula Mariana Veliz, Gabriela Rivas-Mariño, Carina Vance Mafla, Luz María Martínez Altamirano, Cecilia Vaca Jones. 2017. "Etiquetado de Alimentos en Ecuador: Implementación, Resultados y Acciones Pendientes." *Revista Panamericana de Salud Pública* 41: e54.	Ecuador	Non-fragile (MIC)	Case study
40	Diet	Lainie Rutkow, Jesse Jones-Smith, Hannah J. Walters, Marguerite O'Hara, and Sara N. Bleich. 2016. "Factors That Encourage and Discourage Policy-Making to Prevent Childhood Obesity: Experience in the United States." Journal of Public Health Policy 37: 514–527. https://doi.org/10.1057/s41271-016-0035-y	USA	Non-fragile (HIC)	Primary qualitative: KIIs
41	Diet	Sirinya Phulkerd, Stefanie Vandevijvere, Mark Lawrence, Viroj Tangcharoensathien, and Gary Sacks. 2017. "Level of Implementation of Best Practice Policies for Creating Healthy Food Environments: Assessment by State and Non-State Actors in Thailand." *Public Health Nutrition* 20 (3): 381–390. doi:10.1017/S1368980016002391	Thailand	Non fragile (MIC)	Mixed method
42	Diet	Donley Studlar and Paul Cairney. 2019. "Multilevel Governance, Public Health and the Regulation of Food: Is Tobacco Control Policy a Model?" *Journal of Public Health Policy* 40: 147–165. https://doi.org/10.1057/s41271-019-00165-6	UK, USA	Non-fragile (HIC)	Document analysis
43	Diet	Anne Marie Thow, Wendy Snowdon, Ronald Labonté, Deborah Gleeson, David Stuckler, Libby Hattersley, Ashley Schram, Adrian Kay, and Sharon Frielf. 2015. "Will the Next Generation of Preferential Trade and Investment Agreements Undermine Prevention of Noncommunicable Diseases? A Prospective Policy Analysis of the Trans Pacific Partnership Agreement." Health Policy 119 (1): 88–96. https://doi.org/10.1016/j.healthpol.2014.08.002	countries involved in trans-Pacific Partnership Agreement (TPPA)	Global	Policy analysis
44	Diet	Barbara Von Tigerstrom. 2013. "How Do International Trade Obligations Affect Policy Options for Obesity Prevention? Lessons from Recent Developments in Trade and Tobacco Control." *Canadian Journal of Diabetes* 37 (3): 182–188	WTO	Global	Document analysis
45	Diet	Boyd Swinburn, Vivica Kraak, Harry Rutter, Stefanie Vandevijvere, Tim Lobstein, Gary Sacks, Fabio Gomes, Tim Marsh, and Roger Magnusson. 2015. "Strengthening of Accountability Systems to Create Healthy Food Environments and Reduce Global Obesity." *Lancet* 385 9986.: 2534–2545.	Global	Global	Case study

46	Diet	Dariush Mozaffarian, Sonia Y. Angell, Tim Lang, and Juan A. Rivera. 2018. “Role of Government Policy in Nutrition—Barriers to and Opportunities for Healthier Eating.” *BMJ* 361: k2426. doi:10.1136/bmj.k2426	global	Global	Policy analysis
47	Diet	Anne Marie Thow, David Sanders, Eliza Drury, Thandi Puoane, Syeda N. Chowdhury, Lungiswa Tsolekile, and Joel Negin. 2015. “Regional Trade and the Nutrition Transition: Opportunities to Strengthen NCD Prevention Policy in the Southern African Development Community.” Global Health Action 8: 1. doi: 10.3402/gha.v8.28338	15 countries in Southern African Development Community	Non-fragile (LMIC)	Policy analysis
48	Diet, tobacco	R. S. Magnusson. 2015. “Case Studies in Nanny State Name-Calling: What Can We Learn?” Public Health 129 (8): 1074–1082. https://doi.org/10.1016/j.puhe.2015.04.023	Global	Global	Case study
49	Fat	Uriyoán Colón-Ramos, Rafael Monge-Rojas, and Hannia Campos. 2014. “Impact of WHO Recommendations to Eliminate Industrial Trans-Fatty Acids from the food supply in Latin America and the Caribbean.” Health Policy and Planning 29 (5): 529–541. https://doi.org/10.1093/heapol/czt034	13 countries in Latin America and the Caribbean	Non-fragile (MIC)	Mixed method
50	Fat	Shauna M. Downs, Anne Marie Thow, Suparna Ghosh-Jerath, and Stephen R. Leeder. 2015. “Aligning Food-Processing Policies to Promote Healthier Fat Consumption in India.” Health Promotion International 30 (3): 595–605. https://doi.org/10.1093/heapro/dat094	India	Non-fragile (MIC)	Mixed method
51	Fat	World Health Organization. 2019. Countdown to 2023: WHO Report on Global Trans-Fat Elimination 2019. Washington, DC: World Health Organization. https://apps.who.int/iris/handle/10665/331300.	Global	Global	Case study
52	Hypertension, diabetes	Meena Daivadanam , Maia Ingram, Kristi Sidney Annerstedt, et al, on behalf of the GACD Concepts and Contexts working group. 2019. “The Role of Context in Implementation Research for Non-Communicable Diseases: Answering the ‘How-To’ Dilemma.” PLoS ONE 14 (4): e0214454. https://doi.org/10.1371/journal.pone.0214454	Global	Global	Survey
53	Hypertension, diet	Amos K. Laar, Alma J. Adler, Agnes M. Kotoh, Helena Legido-Quigley, Isabelle L. Lange, Pablo Perel, and Peter Lamptey. 2019. “Health System Challenges to Hypertension and Related Non-Communicable Diseases Prevention and Treatment: Perspectives from Ghanaian Stakeholders.” BMC Health Services Research 19: 693. https://doi.org/10.1186/s12913-019-4571-6	Ghana	Non-fragile (LMIC)	Qualitative method
54	Hypertension, diet	Roger Magnusson and Belinda Reeve. 2015. “Food Reformulation, Responsive Regulation, and "Regulatory Scaffolding": Strengthening Performance of Salt Reduction Programs in Australia and the United Kingdom.” Nutrients 7 (7): 5281–5308. https://doi.org/10.3390/nu7075221	Australia, UK	Non-fragile (HIC)	Document analysis
55	NCD	Salim M. Adib. 2014. Non Communicable Disease Prevention and Control Plan (NCD-PCP) for Lebanon. WHO Lebanon Office. https://extranet.who.int/ncdccs/Data/LBN_B3_Final%20plan%202014.pdf	Lebanon	Non-fragile (MIC)	Case study
56	NCD	Luke N. Allen, Brian D. Nicholson, Beatrice Y.T. Yeung, and Francisco Goiana-da-Silva. 2020. “Implementation of Non-Communicable Disease Policies: A Geopolitical Analysis of 151 Countries.” *Lancet Global Health* 8 (1): e50–e58.	Analysis of 151 countries	Global	Geopolitical analysis
57	NCD	Pepita Barlow, Ronald Labonte, Martin McKee, and David Stuckler. 2018. “Trade Challenges at the World Trade Organization to National Noncommunicable Disease Prevention Policies: A Thematic Document Analysis of Trade and Health Policy Space.” PLoS Medicine 15 (6): e1002590. https://doi.org/10.1371/journal.pmed.1002590	122 WTO member states	Global	Document analysis
58	NCD	Tuhin Biswas, Sonia Pervin, Md. Imtiaz Alam Tanim, Louis Niessen, and Anwar Islam. 2017. “Bangladesh Policy on Prevention and Control of Non-Communicable Diseases: A Policy Analysis.” BMC Public Health 17: 582. https://doi.org/10.1186/s12889-017-4494-2	Bangladesh	Fragile (LMIC)	Policy analysis
59	NCD	Elizabeth L. Budd, Anna J. deRuyter, Zhaoxin Wang, Pauline Sung-Chan, Xiangji Ying, Karishma S. Furtado, Tahna Pettman, Rebecca Armstrong, Rodrigo S. Reis, Jianwei Shi, Tabitha Mui, Tahnee Saunders, Leonardo Becker, and Ross C. Brownson. 2018. “A Qualitative Exploration of Contextual Factors that Influence Dissemination and Implementation of Evidence-Based Chronic Disease Prevention Across Four Countries.” BMC Health Services Research 18: 233. https://doi.org/10.1186/s12913-018-3054-5	Australia, Brazil, China, the US	Non-fragile (HIC + MIC)	Qualitative method
60	NCD	Oyun Chimeddamba, Anna Peeters, Helen L. Walls, and Catherine Joyce. 2015. “Noncommunicable Disease Prevention and Control in Mongolia: A Policy Analysis.” BMC Public Health 15: 660. https://doi.org/10.1186/s12889-015-2040-7	Mongolia	Non-fragile (MIC)	Policy analysis

61	NCD	Yodi Christiani, Paul Dugdale, Meredith Tavener, and Julie E. Byles. 2016. "The Dynamic of Non-Communicable Disease Control Policy in Indonesia." *Australian Health Review* 41: 207–213.	Indonesia	Non-fragile (MIC)	Policy analysis
62	NCD	Rebecca Dodd, Erica Reeve, Emalie Sparks, Anita George, Paula Vivili, Si Thu Win Tin, Dai Buresova, Jacqui Webster, and Anne-Marie Thow. 2020. "The Politics of Food in the Pacific: Coherence and Tension in Regional Policies on Nutrition, the Food Environment and Non-Communicable Diseases." *Public Health Nutrition* 23 (1): 168–180. doi:10.1017/S1368980019002118	Twenty-two Pacific island countries and territories	Fragile (MIC) [not for OECD]	Document analysis
63	NCD	Beverley M. Essue and Lydia Kapiriri. 2018. "The Unfunded Priorities: An Evaluation of Priority Setting for Noncommunicable Disease Control in Uganda." Global Health 14: 22. https://doi.org/10.1186/s12992-018-0324-2	Uganda	Fragile (LIC)	Mixed method
64	NCD	Karishma S. Furtado, et al. 2018. "Exploring Political Influences on Evidence-Based Non-Communicable Disease Prevention Across Four Countries." Health Education Research 33 (2): 89–103. https://doi.org/10.1093/her/cyy005	Australia, Brazil, China and the United States	Non-fragile (HIC + MIC)	Qualitative method
65	NCD	Whitney R. et al, 2018. "Implementation of Policy, Systems, and Environmental Community-Based Interventions for Cardiovascular Health Through a National Not-for-Profit: A Multiple Case Study." Health Education and Behavior 45 (6): 855-864. doi:10.1177/1090198118770489	USA	Non-fragile (HIC)	Case study
66	NCD	Anne C. Grunseit, Samantha Rowbotham, Melanie Crane, Devon Indig, Adrian E. Bauman, and Andrew Wilson. 2019. "Nanny or Canny? Community Perceptions of Government Intervention for Preventive Health." Critical Public Health 29 (3): 274–289. doi: 10.1080/09581596.2018.1468020	Australian states (New South Wales, Victoria, Tasmania)	Non-fragile (HIC)	Mixed method
67	NCD	Pamela A. Juma, Shukri F. Mohamed, Beatrice L. Matanje Mwagomba, Catherine Ndinda, Clarisse Mapa-tassou, Mojisola Oluwasanu, Oladimeji Oladepo, Opeyemi Abiona, Misheck J. Nkhata, Jennifer P. Wisdom, and Jean-Claude Mbanya. 2018. "Non-Communicable Disease Prevention Policy Process in Five African Countries Authors." BMC Public Health 18: 961. https://doi.org/10.1186/s12889-018-5825-7	Kenya, South Africa, Cameroon, Nigeria, Malawi	Fragile (LIC) + Non-fragile (MIC)	Case study
68	NCD	Vivian Lin, Catherine Jones, Shiyong Wang, and Enis Baris. 2014. Health in All Policies as a Strategic Policy Response to NCDs. Health, Nutrition, and Population (HNP) discussion paper. World Bank, Washington, DC. https://openknowledge.worldbank.org/handle/10986/20064	Global case studies	Global	Document analysis and case study
69	NCD	WHO Regional Office for Western Pacific. 2012. Regional Meeting on National Multisectoral Plans for NCD Prevention and Control. Manila : WHO Regional Office for the Western Pacific. http://iris.wpro.who.int/handle/10665.1/12558	WPR	Non-fragile (HIC + MIC)	Meeting report
70	NCD	Johan P. MacKenbach and Martin McKee. 2013. "Social-Democratic Government and Health Policy in Europe: A Quantitative Analysis." International Journal of Health Services 43 (3): 389–413. doi:10.2190/HS.43.3.b	Europe	Non-	Quantitative method
71	NCD	Roger S. Magnusson and David Patterson. 2014. "The Role of Law and Governance Reform in the Global Response to Non-Communicable Diseases." Global Health 10: 44. https://doi.org/10.1186/1744-8603-10-44	Global	Global	Document analysis
72	NCD	Roger S. Magnusson, Benn McGrady, Lawrence Gostin, David Patterson, and Hala Abou Taleb. 2019. "Legal Capacities Required for Prevention and Control of Noncommunicable Diseases." Bulletin of the World Health Organization 97 (2): 108–117. http://dx.doi.org/10.2471/BLT.18.213777	Global (India, Uganda, Colombia, USA, Chile, Mexico, SA, UK)	Global [Fragile (LIC) + Non-fragile (HIC/MIC)]	Document analysis
73	NCD	Heather Manson, Terrence Sullivan, Phat Ha, Christine Navarro, and José M. Martín-Moreno. 2013. "Goals Are Not Enough: Building Public Sector Capacity for Chronic Disease Prevention." Public Health Review 35: 11. https://doi.org/10.1007/BF03391696	Global	Global	Case study

74	NCD	Juma, P; Mohamed S & Kyobutungi C. 2017. Analysis of non-communicable disease prevention policies in Kenya. Nairobi: APHRC, http://hdl.handle.net/10625/57553	Kenya	Non-Fragile (LMIC)	Qualitative method (case study)
75	NCD	Shanthi Mendis and Oleg Chestnov. 2013. "Policy Reform to Realize the Commitments of the Political Declaration on Noncommunicable Diseases." British Medical Bulletin 105 (1): 7–27. https://doi.org/10.1093/bmb/ldt001	Global	Global	Mixed method
76	NCD	Mulenga M. Mukanu, Joseph Mumba Zulu, Chrispin Mweemba, and Wilbroad Mutale. 2017. "Responding to Non-Communicable Diseases in Zambia: A Policy Analysis." Health Research Policy and Systems 15: 34. https://doi.org/10.1186/s12961-017-0195-7	Zambia	Fragile (LMIC)	Case study
77	NCD	Catherine Ndinda, Tidings P. Ndhlovu, Pamela Juma, Gershim Asiki, and Catherine Kyobutungi. 2018. "The Evolution of Non-Communicable Diseases Policies in Post-Apartheid South Africa." BMC Public Health 18: 956. https://doi.org/10.1186/s12889-018-5832-8]	South Affrica	Non-fragile (MIC)	Document analysis
78	NCD	Suladda Pongutta, Rapeepong Suphanchaimat, Walaiporn Patcharanarumol, and Viroj Tangcharoensathien. 2019. "Lessons from the Thai Health Promotion Foundation." Bulletin of the World Health Organization 97 (3): 213–220. https://doi.org/10.2471/BLT.18.220277	Thailand	Non-fragile (MIC)	Document analysis
79	NCD	Linda Richter-Sundberg, Therese Kardakis, Lars Weinehall, Rickard Garvare, and Monica E Nyström. 2015. "Addressing Implementation Challenges During Guideline Development–A Case Study of Swedish National Guidelines for Methods of Preventing Disease." BMC Health Services Research 15: 19. https://doi.org/10.1186/s12913-014-0672-4	Sweden	Non-fragile (HIC)	Qualitative method (case study)
80	NCD	Jianwei Shi, Leiyu Shi, Jinsong Geng, Rui Liu, Xin Gong, Xiaojie Bo, Ning Chen, Qian Liu, Yan Yang, and Zhaoxin Wang. 2019. "Status of Evidence-Based Chronic Diseases Prevention Implementation in Shanghai, China: A Qualitative Study." International Journal of Health Planning and Management 34 (3): 912–925. https://doi.org/10.1002/hpm.2863	China	Non-fragile (UMIC)	Qualitative method
81	NCD	Gyambo Sithey, Mu Li, and Anne Marie Thow. 2018. "Strengthening Non-Communicable Disease Policy with Lessons from Bhutan: Linking Gross National Happiness and Health Policy Action." Journal of Public Health Policy 39: 327–342. https://doi.org/10.1057/s41271-018-0135-y	Bhutan	Non-fragile (LMIC)	Document analysis and interviews
82	NCD	Titiporn Tuangratananon, Sangay Wangmo, Nimali Widanapathirana, Suladda Pongutta, Shaheda Viriyathorn, Walaiporn Patcharanarumol, Kouland Thin, Somil Nagpal, Christian Edward L. Nuevo, Retna Siwi Padmawati, Maria Elizabeth Puyat-Murga, Laksono Trisnantoro, Kinzang Wangmo, Nalinda Wellappuli, Phuong Hoang Thi, Tuan Khuong Anh, Thinley Zangmo, and Viroj Tangcharoensathien. 2019. "Implementation of National Action Plans on Noncommunicable Diseases, Bhutan, Cambodia, Indonesia, Philippines, Sri Lanka, Thailand and Viet Nam." Bulletin of the World Health Organization 97 (2): 129–141. https://doi.org/10.2471/BLT.18.220483	Bhutan, Cambodia, Indonesia, Philippines, Sri Lanka, Thailand and Viet Nam.	Non-fragile (MIC)	Document analysis and interviews
83	NCD	Nigel Unwin, T. Alafia Samuels, Trevor Hassell, Ross C. Brownson, and Cornelia Guell. 2017. "The Development of Public Policies to Address Non-Communicable Diseases in the Caribbean Country of Barbados: The Importance of Problem Framing and Policy Entrepreneurs." International Journal of Health Policy and Management 6 (2): 71–82. https://doi.org/10.15171/ijhpm.2016.74	Barbados	Non-fragile (HIC)	Mixed method
84	NCD	Baktygul Akkazieva, Juan Tello, Barton Smith, Melitta Jakab, Konstantin Krasovsky, Nina Sautenkova, Lola Yuldasheva, and Mekhre Shoismatyuloeva .2015 .Better Non-Communicable Disease Outcomes :Challenges and Opportunities for Health Systems .Tajikistan Country Assessment .Regional Office for Europe, World Health Organization, Copenhagen. https://apps.who.int/iris/handle/10665/153907	Tajikistan	Non-fragile (LIC)	Case study
85	NCD	Yong Ho Khang. 2013. "Burden of Noncommunicable Diseases and National Strategies to Control Them in Korea." Journal of Preventive Medicine and Public Health 46 (4): 155–164. https://doi.org/10.3961/jpmph.2013.46.4.155	South Korea	Non-fragile (LIC)	Case study
86	NCD	Regional Office for the Western Pacific. 2017. Progress on the Prevention and Control of Noncommunicable Diseases in the Western Pacific Region: Country Capacity Survey. Regional Office for the Western Pacific, World Health Organization, Manila. http://iris.wpro.who.int/handle/10665.1/14162	WPR	Fragile+ Non-fragile (HIC+MIC)	Survey

87	NCD, medicines	Jordan D. Jarvis, Hannah Woods , Anjli Bali , Efosa Oronsaye, and Nav Persaud. 2019. "Selection of WHO-Recommended Essential Medicines for Non-Communicable Diseases on National Essential Medicines Lists." PLoS ONE 14 (8): e0220781. https://doi.org/10.1371/journal.pone.0220781	China	Non-fragile (UMIC)	Qualitative method
88	Obesity	Lucy C. Farrell, Vivienne M. Moore, Megan J. Warin, and Jackie M. Street. 2019. "Why Do the Public Support or Oppose Obesity Prevention Regulations? Results from a South Australian Population Survey." Health Promotion Journal of Australia 30 (1): 47–59. https://doi.org/10.1002/hpja.185	Bhutan	Non-fragile (LMIC)	Document analysis and interviews
89	Diet	Mohammad Amerzadeh and Amirhossein Takian. 2020. "Reducing Sugar, Fat, and Salt for Prevention and Control of Noncommunicable Diseases (NCDs) as an Adopted Health Policy in Iran." Medical Journal of the Islamic Republic of Iran 34: 136. https://doi.org/10.34171/mjiri.34.136	Iran	Fragile (UMIC)	Mixed method
90	Physical activity	Rodney S. Lyn, Erica R. Sheldon, and Michael P. Eriksen. 2017. "Adopting State-Level Policy to Support Physical Activity Among School-Aged Children and Adolescents: Georgia's SHAPE Act." Public Health Reports 132 (2_suppl): 9S–15S. doi:10.1177/0033354917719705	Australia	Non-fragile (HIC)	Survey
91	Physical activity	Madhuvanti M. Murphy, Nigel Unwin, T. Alafia Samuels, Trevor A Hassell, Lisa Bishop, and Cornelia Guell. 2018. "Evaluating Policy Responses to Noncommunicable Diseases in Seven Caribbean Countries: Challenges to Addressing Unhealthy Diets and Physical Inactivity." Pan American Journal of Public Health 42: e174. https://doi.org/10.26633/RPSP.2018.174	Caribbean region	Non-fragile (HIC + MIC)	Qualitative method
92	Physical activity	Christopher E. Politis, David L. Mowat, and Deb Keen. 2017. "Pathways to Policy: Lessons Learned in Multisectoral Collaboration for Physical Activity and Built Environment Policy Development from the Coalitions Linking Action and Science for Prevention (CLASP) Initiative." Canadian Journal of Public Health 108 (2): e192–e198. https://doi.org/10.17269/cjph.108.5758	Canada	Non-fragile (HIC)	Qualitative method
93	Physical activity	Katie A. Weatherson, Rhyann McKay, Heather L. Gainforth, and Mary E. Jung. 2017. "Barriers and Facilitators to the Implementation of a School-Based Physical Activity Policy in Canada: Application of the Theoretical Domains Framework." BMC Public Health 17: 83. https://doi.org/10.1186/s12889-017-4846-y	Canada	Non-fragile (HIC)	Qualitative method
94	Physical activity, diet	Saskia Muellmann, Berit Steenbock, Katrien De Cocker, Marieke De Craemer, Catherine Hayes, Miriam P. O'Shea, Karolina Horodyska, Justyna Bell, Aleksandra Luszczynska, Gun Roos, Lars Jørun Langøien, Gro Rugseth, Laura Terragni, Ilse De Bourdeaudhuij, Johannes Brug, and Claudia R. Pischke. 2017. "Views of Policy Makers and Health Promotion Professionals on Factors Facilitating Implementation and Maintenance of Interventions and Policies Promoting Physical Activity and Healthy Eating: Results of the DEDIPAC Project." BMC Public Health 17: 932. https://doi.org/10.1186/s12889-017-4929-9	Europe	Non-fragile (HIC)	Mixed method
95	Physical activity, diet	Mojisola Oluwasanu, Opeyemi Oladunni, amd Oladimeji Oladepo. 2020. "Multisectoral Approach and WHO 'Bestbuys' in Nigeria's Nutrition and Physical Activity Policies." Health Promotion International 35 (6): 1383–1393. https://doi.org/10.1093/heapro/daaa009	Nigeria	Fragile (LMIC)	Case study
96	Physical activity	Fiona Bull, Karen Milton, Sonja Kahlmeier, Alberto Arlotti, Andrea Backović Juričan, Olov Belander, Brian Martin, Eva Martin-Diener, Ana Marques, Jorge Mota, Tommi Vasankari, and Anita Vlasveld. 2015. "Turning the Tide: National Policy Approaches to Increasing Physical Activity in Seven European Countries." *British Journal of Sports Medicine* 49: 749–56.	Finland, Italy, the Netherlands, Norway, Portugal, Slovenia, Switzerland	Non-fragile (HIC)	Policy analysis
97	Salt	Adriana Blanco-Metzler, et al 2012. "Avances en la reducción del consumo de sal y sodio en Costa Rica [Advances in Reducing Salt and Sodium Intake in Costa Rica]. Revista Panamericana de Salud Publica 32 (4): 316–320. https://doi.org/10.1590/s1020-49892012001000011	Costa Rica	Non-fragile (UMIC)	Document analysis

98	Salt	Norm Campbell, Barbara Legowski, Branka Legetic, Daniel Ferrante, Eduardo Nilson, Christine Campbell, and Mary L'Abbé. 2014. "Targets and Timelines for Reducing Salt in Processed Food in the Americas." Journal of Clinical Hypertension 16 (9): 619–623. https://doi.org/10.1111/jch.12379	8 countries in the Americas region	Non-fragile (HIC + MIC)	Policy analysis
99	Salt	Luciana Castronuovo,et al. 2017. "Analysis of a Voluntary Initiative to Reduce Sodium in Processed and Ultra-Processed Food Products in Argentina: The Views of Public and Private Sector Representatives." Cadernos de Saude Publica 33 (6): e00014316. https://doi.org/10.1590/0102-311X00014316	Argentina	Non-fragile (UMIC)	Qualitative method
100	Salt	Priti Gupta, Sailesh Mohan, Claire Johnson, Vandana Garg, Sudhir Raj Thout, Roopa Shivashankar, Anand Krishnan, Bruce Neal, and Dorairaj Prabhakaran. 2018. "Stakeholders' Perceptions Regarding a Salt Reduction Strategy for India: Findings from Qualitative Research." PLoS ONE 13 (8): e0201707. https://doi.org/10.1371/journal.pone.0201707	India	Non-fragile (MIC)	Qualitative method
101	Salt	F. He, H. Brinsden, and G. MacGregor. 2014. "Salt Reduction in the United Kingdom: A Successful Experiment in Public Health." Journal of Human Hypertension 28: 345–352. https://doi.org/10.1038/jhh.2013.105	UK	Non-fragile (HIC)	Policy analysis
102	Salt	Branka Legetic and Norm Campbell. 2011. "Reducing Salt Intake in the Americas: Pan American Health Organization Actions." Journal of Health Communication 16 Suppl 2: 37–48. https://doi.org/10.1080/10810730.2011.601227	America region	Non-fragile (HIC+MIC)	Case study
103	Salt	Shahram Rafieifar, Hamed Pouraram, Abolghassem Djazayery, Fereydoun Siassi, Zahra Abdollahi, Ahmad Reza Dorosty, Mitra Abtahi, Hossein Kazemeini, and Farshad Farzadfar. 2016. "Strategies and Opportunities Ahead to Reduce Salt Intake." *Archives of Iranian Medicine* 19 (10): 729–734.	Iran	Fragile (UMIC)	Document analysis
104	Salt	Belinda Reeve and Roger Magnusson. 2015. "Food Reformulation and the (Neo)-Liberal State: New Strategies for Strengthening Voluntary Salt Reduction Programs in the UK and USA." *Public Health* 129 (8): 1061–1073	UK, USA	Non-fragile (HIC)	Document analysis
105	Salt	Kathy Trieu, Jacqui Webster, Stephen Jan, Silvia Hope, Take Naseri, Merina Ieremia, Colin Bell, Wendy Snowdon, and Marj Moodie. 2018. "Process Evaluation of Samoa's National Salt Reduction Strategy (MASIMA): What Interventions Can Be Successfully Replicated in Lower-Income Countries?" Implementation Science 13: 107. https://doi.org/10.1186/s13012-018-0802-1	Samoa	Non-fragile (UMIC)	Process evaluation and surveys
106	Diet	Amos Laar, Amy Barnes, Richmond Aryeetey, Akua Tandoh, Kristin Bash, Kobby Mensah, Francis Zotor, Stefanie Vandevijvere, and Michelle Holdsworth. 2020. "Implementation of Healthy Food Environment Policies to Prevent Nutrition-Related Non-Communicable Diseases in Ghana: National Experts' Assessment of Government Action." Food Policy 93, 101907. https://doi.org/10.1016/j.foodpol.2020.101907	Ghana	Non-fragile (LMIC)	Policy analysis
107	Salt	Karen Charlton, Jacqui Webster, and Paul Kowal. 2014. "To Legislate or Not to Legislate? A Comparison of the UK and South African Approaches to the Development and Implementation of Salt Reduction Programs." Nutrients 6 (9): 3672–3695. https://doi.org/10.3390/nu6093672	UK and South Africa	Non-fragile (HIC + MIC)	Case study
108	Sugar	Carlos M. Guerrero-López, Mishel Unar-Munguía, and M. Arantxa Colchero. 2017. "Price Elasticity of the Demand for Soft Drinks, Other Sugar-Sweetened Beverages and Energy Dense Food in Chile." BMC Public Health 17: 180. https://doi.org/10.1186/s12889-017-4098-x	Mexico	Non-fragile (UMIC)	Quantitative method
109	Sugar	Susan Greenhalgh. 2019. "Soda Industry Influence on Obesity Science and Policy in China." Journal of Public Health Policy 40: 5–16. https://doi.org/10.1057/s41271-018-00158-x	China	Non-fragile (UMIC)	Qualitative method
110	Sugar	Alex Myers, David Fig, Aviva Tugendhaft, Jonathan E. Myers, and Karen J. Hofman 2017. "The History of the South African Sugar Industry Illuminates Deeply Rooted Obstacles for Sugar Reduction Anti-Obesity Interventions." African Studies 76 (4): 475–490. doi: 10.1080/00020184.2017.1311515	South Africa	Non-fragile (UMIC)	Case study
111	Sugar	Frances Onagan, Beverly Ho, and Karl Kendrick Chua. 2019. "Development of a Sweetened Beverage Tax, Philippines." Bulletin of the World Health Organization 97 (2): 154–159. https://doi.org/10.2471/BLT.18.220459	Philippines	Non-fragile (UMIC)	Document analysis

112	Sugar	Vicente Ortún, Beatriz G López-Valcárcel, and Jaime Pinilla. 2016. "Tax on Sugar Sweetened Beverages in Spain." *Revista Española de Salud Pública* 13 (90) :e1-e13.	Multi-country (Spain, Mexico)	Non-fragile (HIC + MIC)	Document analysis
113	Sugar	Christina A. Roberto and Jennifer L. Pomeranz. 2015. "Public Health and Legal Arguments in Favor of a Policy to Cap the Portion Sizes of Sugar-Sweetened Beverages." American Journal of Public Health 105 (11): 2183–2190. https://doi.org/10.2105/AJPH.2015.302862	New York, US	Non-fragile (HIC)	Document analysis
114	Sugar	Alex Myers, David Fig, Aviva Tugendhaft, Jessie Mandle, Jonathan Myers, and Karen Hofman. 2017. "Sugar and Health in South Africa: Potential Challenges to Leveraging Policy Change." Global Public Health 12 (1): 98–115. doi: 10.1080/17441692.2015.1071419	South Africa	Non-fragile (UMIC)	Case study
115	Tobacco	Naowarut Charoenca, Jeremiah Mock, Nipapun Kungskulniti, Sunida Preechawong, Nicholas Kojetin, and Stephen L. Hamann. 2012. "Success Counteracting Tobacco Company Interference in Thailand: An Example of FCTC Implementation for Low- and Middle-income Countries." International Journal of Environmental Researchand Public Health 9: 1111–1134. https://doi.org/10.3390/ijerph9041111	Thailand	Non-fragile (MIC)	Qualitative method
116	Tobacco	Jeff Collin, Sarah E. Hill, Mor Kandlik Eltanani, Evgeniya Plotnikova, Rob Ralston, and Katherine E. Smith. 2017. "Can Public Health Reconcile Profits and Pandemics? An Analysis of Attitudes to Commercial Sector Engagement in Health Policy and Research." PLoS ONE 12 (9): e0182612. https://doi.org/10.1371/journal.pone.0182612	40 countries (HIC and LMIC)	Global	Survey
117	Tobacco	Eric Crosbie, George Thomson, Becky Freeman, and Stella Bialous. 2018. "Advancing Progressive Health Policy to Reduce NCDs Amidst International Commercial Opposition: Tobacco Standardised Packaging in Australia." Global Public Health 13 (12): 1753–1766. doi: 10.1080/17441692.2018.1443485	Australia	Non-fragile (HIC)	Mixed method
118	Tobacco	Vera Luiza da Costa e Silva, Daniela Pantani, Mônica Andreis, Robert Sparks, and Ilana Pinsky. 2013. "Bridging the Gap Between Science and Public Health: Taking Advantage of Tobacco Control Experience in Brazil to Inform Policies to Counter Risk Factors for Non-Communicable Diseases." Addiction 108 (8): 1360–1366. https://doi.org/10.1111/add.12203	Brazil	Non-fragile (UMIC)	Case study
119	Tobacco	Amanda Fallin, Amie Goodin, Mary Kay Rayens, Sarah Morris, and Ellen J. Hahn. 2014. "Smoke-Free Policy Implementation: Theoretical and Practical Considerations." Policy, Politics, and Nursing Practice 15 (3-4): 81–92. doi:10.1177/1527154414562301	USA	Non fragile (HIC)	Document analysis
120	Tobacco	Soumita Ghose, Alok Sardar, Suman Shiva, Brega Ellen Mullan, and Soumitra S Datta. 2019. "Perception of Tobacco Use in Young Adults in Urban India: A Qualitative Exploration with Relevant Health Policy Analysis." Ecancermedicalscience 13: 915. https://doi.org/10.3332/ecancer.2019.915	India	Non-fragile (LMIC)	Qualitative method
121	Tobacco	Holly Jarman. 2013. "Attack on Australia: Tobacco Industry Challenges to Plain Packaging." Journal of Public Health Policy 34: 375–387. https://doi.org/10.1057/jphp.2013.18	Australia	Non-fragile (HIC)	Document analysis
122	Tobacco	Ronald Labonté, Raphael Lencucha, Fastone Goma, Richard Zulu, and Jeffrey Drope. 2019. "Consequences of Policy Incoherence: How Zambia's Post-FCTC Investment Policy Stimulated Tobacco Production." Journal of Public Health Policy 40 (3): 286–291. https://doi.org/10.1057/s41271-019-00171-8	Zambia	Fragile (LMIC)	Qualitative method
123	Tobacco	Jennifer Leeman, Allison Myers, Jennifer C. Grant, Mary Wangen, and Tara L. Queen. 2017. "Implementation Strategies to Promote Community-Engaged Efforts to Counter Tobacco Marketing at the Point of Sale." Translational Behavioral Medicine 7 (3): 405–414. https://doi.org/10.1007/s13142-017-0489-x	USA	Non-fragile (HIC)	Qualitative method
124	Tobacco	Raphael Lencucha, Jeffrey Drope, and Jenina Joy Chavez. 2015. "Whole-of-Government Approaches to NCDs: The Case of the Philippines Interagency Committee-Tobacco." Health Policy and Planning 30 (7): 844–852. https://doi.org/10.1093/heapol/czu085	Philippines	Non-fragile (MIC)	Mixed method

125	Tobacco	Clarisse Mapa-Tassou, Cecile Rénée Bonono, Felix Assah, Jennifer Wisdom, Pamela A. Juma, Jean-Claude Katte, Zakariaou Njoumemi, Pierre Ongolo-Zogo, Leopold K. Fezeu, Eugene Sobngwi, and Jean Claude Mbanya. 2018. "Two Decades of Tobacco Use Prevention and Control Policies in Cameroon: Results from the Analysis of Non-Communicable Disease Prevention Policies in Africa." BMC Public Health 18: 958 2018. https://doi.org/10.1186/s12889-018-5828-4	Cameroon	Fragile (LMIC)	Case study
126	Tobacco	Lazarous Mbulo, et al on behalf of GATS collaborative group. 2016. "Preventing Tobacco Epidemic in LMICs with Low Tobacco Use—Using Nigeria GATS to review WHO MPOWER Tobacco Indicators and Prevention Strategies." Preventive Medicine 91S: S9–S15. https://doi.org/10.1016/j.ypmed.2016.04.005	Nigeria	Fragile (LMIC)	Survey
127	Tobacco	Ashleigh Cussen and Judith McCool. 2011. "Tobacco Promotion in the Pacific: The Current State of Tobacco Promotion Bans and Options for Accelerating Progress." Asia Pacific Journal of Public Health 23 (1): 70–78. doi:10.1177/1010539510390925	WPR	Fragile (MIC) [not for OECD]	Document analysis
128	Tobacco	Judith McCool, et al. 2013. "Supporting Pacific Island Countries to Strengthen Their Resistance to Tobacco Industry Interference in Tobacco Control: A Case Study of Papua New Guinea and Solomon Islands." International Journal of Environmental Research and Public Health 10 (8): 3424–3434. https://doi.org/10.3390/ijerph10083424	Papua New Guinea,Solomon Islands	Fragile (MIC)	Document analysis
129	Tobacco	Shukri F. Mohamed, Pamela Juma, Gershim Asiki, and Catherine Kyobutungi. 2018. "Facilitators and Barriers in the Formulation and Implementation of Tobacco Control Policies in Kenya: A Qualitative Study." BMC Public Health 18: 960. https://doi.org/10.1186/s12889-018-5830-x	Kenya	Fragile (LMIC)	Case study
130	Tobacco	Oladimeji Oladepo, Mojisola Oluwasanu, and Opeyemi Abiona. 2018. "Analysis of Tobacco Control Policies in Nigeria: Historical Development and Application of Multi-Sectoral Action." BMC Public Health 18: 959 2018. https://doi.org/10.1186/s12889-018-5831-9	Nigeria	Fragile (LMIC)	Case study
131	Tobacco	Hilal Ozcebe, Toker Erguder, Mehmet Balcilar, Pavel Ursu, Aaron Reeves, David Stuckler, Andrew Snell, Gauden Galea, Bente Mikkelsen, and Kristina Mauer-Stender. 2018. "The Perspectives of Politicians on Tobacco Control in Turkey." European Journal of Public Health 28 (suppl_2): 17–21. https://doi.org/10.1093/eurpub/cky152	Turkey	Non-fragile (UMIC)	Mixed method
132	Tobacco	Jalal Poorolajal, Younes Mohammadi, and Azam Mahmoodi. 2017. "Challenges of Tobacco Control Program in Iran." *Archives of Iranian Medicine* 20 (4): 229–234.	Iran	Fragile (UMIC)	Survey
133	Tobacco	Erica Cavalcanti Rangel, et al. 2017. "The Decision-Making Process in Brazil's Ratification of the World Health Organization Framework Convention on Tobacco Control." Cadernos de Saude Publica 33 (Suppl 3): e00126115. https://doi.org/10.1590/0102-311X00126115	Brasil	Non-fragile (UMIC)	Qualitative method
134	Tobacco	Lindsay Robertson, Louise Marsh, Janet Hoek, Rob McGee, and Richard Egan. 2015. "Regulating the Sale of Tobacco in New Zealand: A Qualitative Analysis of Retailers' Views and Implications for Advocacy." International Journal on Drug Policy 26 (12): 1222–1230. https://doi.org/10.1016/j.drugpo.2015.08.015	New Zealand	Non-fragile (HIC)	Qualitative method
135	Tobacco	Michal Stoklosa and Hana Ross. 2014. "Tobacco Control Funding for Low-Income and Middle-Income Countries in a Time of Economic Hardship." *Tobacco Control* 23: e122–e126.	LMICs	Global	Case study
136	Tobacco	Saliyou Sanni, Charles Hongoro, Catherine Ndinda, and Jennifer P. Wisdom. 2018. Assessment of the Multi-Sectoral Approach to Tobacco Control Policies in South Africa and Togo. BMC Public Health 18: 962. https://doi.org/10.1186/s12889-018-5829-3	South Africa and Togo	Fragile (LIC) + Non-fragile (HIC/MIC)	Document analysis
137	Tobacco	Travis D. Satterlund, Diana Cassady, Jeanette Treiber, and Cathy Lemp. 2011. "Barriers to Adopting and Implementing Local-Level Tobacco Control Policies." Journal of Community Health 36 (4): 616–623. https://doi.org/10.1007/s10900-010-9350-6	California, USA	Non-fragile (HIC)	Document analysis

138	Tobacco	World Health Organization and WHO Framework Convention on Tobacco Control. 2020. WHO FCTC Implementation Review in Pacific Island Countries. Geneva: World Health Organization. https://apps.who.int/iris/handle/10665/337194.	Pacific island countries	Fragile (MIC) [not for OECD]	Case study
139	Tobacco	Jennifer P et al 2018. Influence of the WHO Framework Convention on Tobacco Control on Tobacco Legislation and Policies in Sub-Saharan Africa, BMC Public Health 18: 954 2018. https://doi.org/10.1186/s12889-018-5827-5	Cameroon, Kenya, Nigeria, Malawi, South Africa, Togo	Fragile (LIC) + Non-fragile (MIC)	Case study
140	Tobacco	World Bank Group. 2018. Reducing Tobacco Use Through Taxation: The Experience of the Republic of Korea. World Bank, Washington, DC. https://openknowledge.worldbank.org/handle/10986/30020	Republic of Korea	Non fragile (HIC)	Case study
141	Tobacco	World Bank Group. 2018. Advancing Action on the Implementation of Tobacco Tax Harmonization in the Organization of Eastern Caribbean States Countries. World Bank, Washington, DC. https://openknowledge.worldbank.org/handle/10986/30034	Carribean Island countries	Non-fragile (MIC)	Case study
142	Tobacco	Robert Beaglehole, Ruth Bonita, Derek Yach, Judith Mackay, and K. Srinath Reddy. 2015. "A Tobacco-Free World: A Call to Action to Phase Out the Sale of Tobacco Products by 2040." *Lancet* 385 (9972): 1011–1018	Global	Global	Case study
143	Tobacco	Anna B. Gilmore, Gary Fooks, Jeffrey Drope, Stella Aguinaga Bialous, and Rachel Rose Jackson. 2015. "Exposing and Addressing Tobacco Industry Conduct in Low-Income and Middle-Income Countries." *Lancet* 385 (9972): 1029–1043.	LMICs	Non-fragile (LMIC)	Case study
144	Tobacco	Judith Mackay, Bungon Ritthiphakdee, and K. Srinath Reddy. 2013. "Tobacco Control in Asia." *Lancet* 381 (9877): 1581–1587.	SEARO and WPRO	Non-fragile (HIC+MiC)	Document analysis
145	Tobacco	Gonghuan Yang, Yu Wang, Yiqun Wu, Jie Yang, and Xia Wan. 2015. "The Road to Effective Tobacco Control in China." *Lancet* 385 (9972): 1019–1028.	China	Non-fragile (UMIC)	Case study
146	Tobacco, food, alcohol	Raphael Lencucha and Anne Marie Thow. 2019. "How Neoliberalism Is Shaping the Supply of Unhealthy Commodities and What This Means for NCD Prevention." International Journal of Health Policy and Management 8 (9): 514–520. https://doi.org/10.15171/ijhpm.2019.56	Malawi, Zambia, Philippines	Fragile + Non-fragile	Prospective analysis
147	Trans fatty acids	Andrey Demin, et al. 2020. "Trans Fatty Acid Elimination Policy in Member States of the Eurasian Economic Union: Implementation Challenges and Capacity for Enforcement." Journal of Clinical Hypertension 22 (8): 1328–1337. https://doi.org/10.1111/jch.13945	Europe	Non fragile (HIC)	Case study
148	Primary care	Lwin Lwin Aye, et al 2020. "Experiences from the Pilot Implementation of the Package of Essential Non-Communicable Disease Interventions (PEN) in Myanmar, 2017-18: A Mixed Methods Study." PLoS ONE 15 (2): e0229081. https://doi.org/10.1371/journal.pone.0229081	Myanmar	Fragile (MIC)	Mixed method
149	CVD	Rawlance Ndejjo, Rhoda K. Wanyenze, Fred Nuwaha, Hilde Bastiaens, and Geofrey Musinguzi. 2020. "Barriers and Facilitators of Implementation of a Community Cardiovascular Disease Prevention Programme in Mukono and Buikwe Districts in Uganda Using the Consolidated Framework for Implementation Research." Implementation Science 15: 106. https://doi.org/10.1186/s13012-020-01065-0	Uganda	Fragile (LIC)	Qualitative method
150	NCD	L.O. Gostin, H. Abou-Taleb, S.A. Roache, and A. Alwan. 2017. "Legal Priorities for Prevention of non-Communicable Diseases: Innovations from WHO's Eastern Mediterranean Region." Public Health 144: 4–12. https://doi.org/10.1016/j.puhe.2016.11.001	EMR	Fragile + Non-fragile	Document analysis
151	Physical activity	Bojana Klepac Pogrmilovic, Andrea Ramirez Varela, Michael Pratt, Karen Milton, Adrian Bauman, Stuart J. H. Biddle, and Zeljko Pedisic. 2020. "National Physical Activity and Sedentary Behaviour Policies in 76 Countries: Availability, Comprehensiveness, Implementation, and Effectiveness." International Journal of Behavioral Nutrition and Physical Activity 17: 116. https://doi.org/10.1186/s12966-020-01022-6	Global	Global	Case study

152	Primary care	Luis Orlando Perez. 2013. Uruguay–UY Non Communicable Diseases Prevention Project : P050716–Implementation Status Results Report : Sequence 10 (). Washington, DC: World Bank Group. http://documents.worldbank.org/curated/en/841071468130210742/Uruguay-UY-Non-Communicable-Diseases-Prevention-Project-P050716-Implementation-Status-Results-Report-Sequence-10	Uruguay	Non-fragile (MIC)	Case study
153	Physical activity	Regional Committee for Africa, 70. 2020. Framework for the Implementation of the Global Action Plan on Physical Activity 2018–2030 in the WHO African Region: Report of the Secretariat. Regional Office for Africa, World Health Organization, Brazzaville, Republic of Congo. https://apps.who.int/iris/handle/10665/333737	African Region	Fragile + Non-fragile	Report
154	CVD	World Health Organization. 2020. Technical Package for Cardiovascular Disease Management in Primary Health Care: Healthy-Lifestyle Counselling. World Health Organization, Geneva. https://apps.who.int/iris/handle/10665/260422.	Global	Global	Technical paper
155	CVD	World Health Organization. 2020. Improving Hypertension Control in 3 Million People: Country Experiences of Programme Development and Implementation. World Health Organization, Geneva. https://apps.who.int/iris/handle/10665/336019	Global	Global	Mixed method
156	CVD	Nicole Fraser-Hurt, Shuo Zhang, Dayo Carol Obure, Leausa Take Naseri, Robert Thompsen, Victoria Ieremia-Faasili, and Athena Matalavea. 2020. Care for Hypertension and Other Chronic Conditions in Samoa: Understanding the Bottlenecks and Closing the Implementation Gaps. World Bank, Washington, DC. https://openknowledge.worldbank.org/handle/10986/33256	Samoa	Fragile (MIC) [not for OECD]	Mixed method
157	CVD	Dylan Collins, Tiina Laatikainen, and Jill Farrington. 2020. "Implementing Essential Interventions for Cardiovascular Disease Risk Management in Primary Healthcare: Lessons from Eastern Europe and Central Asia. *BMJ Global Health* 5: e002111.	Eastern Europe and Central Asia	Non-fragile	Qualitative method
