# Supplementary material for: Barriers and Opportunities for WHO ‘Best Buys’ Non-Communicable Disease Policy Adoption and Implementation From a Political Economy Perspective: A Complexity Systematic Review
Source: Int J Health Policy Manag. 2024 Feb 4;13:7989. doi: 10.34172/ijhpm.2023.7989 (PMC11016278; doi:10.34172/ijhpm.2023.7989)
Supplement: Supplementary file 4 — contains Tables S3 and S4. [file ijhpm-13-7989-s004.pdf]

Article title: Barriers and Opportunities for WHO "Best Buys" Non-communicable Disease Policy Adoption and Implementation From a Political Economy Perspective: A Complexity Systematic Review

Journal name: International Journal of Health Policy and Management (IJHPM)

Authors' information: Giulia Loffreda[1]*, Stella Arakelyan[2,1], Ibrahim Bou-Orm[1], Hampus Holmer[3], Luke N. Allen[4], Sophie Witter[1], Alastair Ager[1], Karin Diaconu[1]

[1]NIHR Research Unit of Health in Fragility, Institute for Global Health and Development, Queen Margaret University Edinburgh, Musselburgh, UK.

[2]Advanced Care Research Centre, Usher Institute, University of Edinburgh, Edinburgh, UK.

[3]Department of Global Public Health, Karolinska Institutet, Stockholm, Sweden.

[4]Department of Clinical Research, London School of Hygiene and Tropical Medicine, London, UK.

***Correspondence to:** Giulia Loffreda; Email: gloffreda@qmu.ac.uk

Citation: Loffreda G, Arakelyan S, Bou-Orm I, et al. Barriers and opportunities for WHO "best buys" non-communicable disease policy adoption and implementation from a political economy perspective: a complexity systematic review. Int J Health Policy Manag. 2024;13:7989. doi:10.34172/ijhpm.2023.7989

Supplementary file 4

Table S3. Results of quality appraisal of included studies using MMAT

	Yes		No		Cannot tell	
	n	%	n	%	N	%
**Qualitative Method (n=124)**						
(S.1) Are there clear research questions?	118	95%	0	0%	6	0.04%
(S.2) Do the collected data allow to address the research questions?	98	84%	0	0%	26	15%
(1.1) Is the qualitative approach appropriate to answer the research question?	96	79%	0	0%	28	20%
(1.2) Are the qualitative data collection methods adequate to address the research question?	105	87%	0	0%	19	12%
(1.3) Are the findings adequately derived from the data?	105	87%	0	0%	19	12%
(1.4) Is the interpretation of results sufficiently substantiated by data?	105	87%	0	0%	19	12%
(1.5) Is there coherence between qualitative data sources, collection, analysis and interpretation?	105	87%	0	0%	19	12%
**Quantitative Method (n=14)**						
(S.1) Are there clear research questions?	13	93%	0	0%	1	7%
(S.2) Do the collected data allow to address the research questions?	13	93%	0	0%	1	7%
(2.1) Is there a clear rationale for using a quantitative method?	14	100%	0	0%	0	0%
(2.2) Is there a clear description of data collection tools?	13	92%	0	0%	1	7%

(2.3) Are there complete outcome data?	12	86%	1	7%	1	7%
(2.4) Do the different components of the study adhere to the quality criteria of each tradition of the methods involved?	14	100%	0	0%	0	0%
**Mixed Methods (n=19)**						
(S.1) Are there clear research questions?	14	74%	0	0%	5	26%
(S.2) Do the collected data allow to address the research questions?	14	74%	0	0%	5	26%
(3.1) Is there an adequate rationale for using a mixed method design to address the research question?	14	74%	0	0%	5	26%
(3.2) Are the different components of the study effectively integrated to answer the research question?	16	84%	0	0%	3	16%
(3.3) Are the outputs of the integration of qualitative and quantitative components adequately interpreted?	16	84%	0	0%	3	16%
(3.4) Are divergences and inconsistencies between quantitative and qualitative results adequately addressed?	16	84%	0	0%	3	16%
(3.5) Do the different components of the study adhere to the quality criteria of each tradition of the methods involved?	19	100%	0	0%	0	0%

Table S4. CERQual Summary of Qualitative Findings table – Summary of key issues

Summary of review findings	Studies contributing to the review finding	CERQual assessment of confidence in the evidence	Explanation of CERQual assessment
**Tobacco**			
Legal capacity was a key resource, often not available, to ensure effective adoption and implementation; in this regard FCTC was reported as an essential tool to catalyse and advance tobacco control, by providing a strong legal framework, political engagement and by accelerating conformity to international standards	12, 67, 71, 73, 75, 82, 90, 92, 115, 116, 117, 118, 121, 122, 125, 126, 129, 130, 131, 139, 140, 143, 145, 146, 154	**High confidence**	Studies have minor methodological limitations. Data from several settings (HIC, MIC, LIC, fragile countries) providing a relatively well generalisable finding. No or very minor concerns about coherence, relevance, and adequacy.
National and regional coordination and collaboration can support countries in the policy process. Such collaboration should not include tobacco industry, as outlined in article 5.3 of FCTC. Involvement of all relevant ministries and organizations is essential.	115, 117, 118, 122, 125, 129, 139, 143, 145, 146,	**Moderate confidence**	Studies have minor methodological limitations. Data is from several settings, including several African countries, providing minor concerns about relevance. Moderate concerns about adequacy. No concerns about relevance.
**Diet**			
Indicators, standards, and accountability mechanisms (including conflict of interest frameworks, guidelines on engagement, monitoring for self-regulations and voluntary measures) are crucial for policy success. Strong government leadership, multisectoral and stakeholder engagement are necessary conditions for strengthening the performance of voluntary or non-statutory food reformulation initiatives.	31, 33, 35, 36, 24, 27, 29, 30, 31, 33, 43, 45, 51, 54, 164, 97, 98, 99, 100, 101, 102, 103, 104, 105, 107, 147, 154	**High confidence**	Studies have minor methodological limitations. Data is from several settings, providing minor concerns about relevance. No concerns about coherence and adequacy.

Knowledge on trade rules is required to implement policies on front of package labelling. Policies need to be implemented within the trade rules and need to demonstrate that policies are the least trade-restricting measure; multinational companies have a great influence because of their economic power, government lobbying and communication and marketing resources. Trade agreements might reduce the scope for governments to implement innovative measures (that have only limited evidence for their effectiveness).	37, 42, 43, 44, 45, 46, 47, 51, 110, 114	**High confidence**	Minor methodological limitations. High coherence, relevance, and adequacy of data.
Limited local evidence and data, lack of research uptake of study findings, no data on food content, consumption, and labelling, perception of weak evidence for the link between diet (e.g., SSBs, TSA, etc) and NCDs, lack of local evidence on interventions effect (e.g., SSB) do not support politicians' commitments to agenda setting and policy development.<br><br>A solid body of evidence is essential to assess the impact of various measures and recommended actions, including evidence on health diets definitions, health and risk distributions among populations, environmental and social values.	103, 24, 26, 29, 30, 33, 35, 36, 38, 40, 43, 44, 45, 46, 49, 50, 51, 52, 54,97, 98, 99, 105, 26, 27, 29, 30, 31, 36, 40, 41, 67, 100, 101, 102, 104, 107, 148	**High confidence**	Minor methodological limitations. High coherence, relevance, and adequacy of data.
**Alcohol**			
Social acceptability of alcohol and culture around it do shape use and prevalence of social norms. In fact, public opposition and competing interests can interfere with policy processes. Consumers can oppose policy,	2, 3, 4, 5, 6, 7, 8, 9, 10	**Moderate confidence**	Minor methodological limitations and moderate concerns regarding adequacy and relevance of data

especially on pricing and shortened licensing hours. Lack of coherence in messaging around policy and appropriate framing of the problem to create political, social, and moral understanding in line with public beliefs and attitudes can help the policy process. Media, as well as civil society play a role in supporting such effort. Community engagement and multisectoral collaboration enable not only better support and accountability.			
**Physical activity**			
Physical activity in many countries has received little political attention. In contexts plagued by ongoing conflicts and instability, emerging and recurring epidemics, making the case for physical activity becomes difficult. This requires strong political will and robust scientific evidence of its health benefits. In many low- and middle-income countries there is still a lack of country and context specific research on PA and health, which could be another reason for lower interest of policymakers to support the promotion of PA.	93, 94, 96, 151, 153	**Moderate confidence**	Minor methodological limitations and moderate concerns regarding adequacy and relevance of data. Minor concerns regarding coherence.
**Clinical guidelines**			
Multiple components need to be considered to reach effective implementation of clinical guidelines, including assessment of the national protocols and adaptation of WHO PEN protocols to the national context, collection of base line indicators, training of staff in pilot PHC units, implementation of interventions and provision of technical support.	13, 15, 18, 20, 23	**Moderate confidence**	Minor methodological limitations and moderate concerns regarding adequacy and relevance of data. Minor concerns regarding coherence.

**Generic issues**			
Technical support from global (e.g., WHO) and regional organizations and collaborations has been proven to be a key facilitator for knowledge sharing for NCD policy development and primary care	13, 15, 16, 18, 81, 86, 143, 151, 154	**High confidence**	Minor methodological limitations. High coherence, relevance, and adequacy of data.
A fragmented governance and the absence of a dedicated structure, with a designated body to oversee planning, guidance, monitoring and evaluation of implementation has been a barrier to effective implementation; involvement from multiple actors without adequate coordination by the MOH created silos and fragmentation in policy and program implementation. A strong governance system that facilitates multisectoral collaboration and partnership building is therefore a prerequisite for any NCD policy process.	1, 11, 12, 24, 27, 30, 31, 33, 54, 55, 60, 61, 65, 67, 68, 69, 71, 73, 75, 82, 83, 84, 85, 94, 97, 100, 104, 105, 107, 111, 115, 117, 118, 119, 123, 130, 145, 146, 151, 154	**High confidence**	Minor methodological limitations. High coherence, relevance, and adequacy of data.
Change perception of problem and solution (e.g., personal responsibility of risk factors) by using the media to increase public visibility of the issue. Increase the public support for the policy (e.g., by organizing a media campaign).	3, 5, 7, 26, 27, 29, 30, 31, 33, 36, 40, 41, 42, 48, 51, 66, 67, 71, 72, 83, 95, 100, 101, 115, 118, 119, 123, 130, 134, 136, 141, 145, 154	**High confidence**	Minor methodological limitations. High coherence, relevance, and adequacy of data.
Define clear accountability frameworks to manage engagement with stakeholders §(particularly with private sectors). Change decision-making processes, in order to prevent some opponents from participating. Map the stakeholders: persuade opponents, seek common goals with supporters and expand their participation.	2, 3, 6, 7, 9, 10, 11, 12, 18, 24, 27, 29, 31, 29, 33, 38, 45, 54, 56, 63, 57, 72, 75, 101, 102, 104, 110, 114, 142	**High confidence**	Minor methodological limitations. High coherence, relevance, and adequacy of data.
